# Schwann cell endosome CGRP signals elicit periorbital mechanical allodynia in mice

**DOI:** 10.1038/s41467-022-28204-z

**Published:** 2022-02-03

**Authors:** Francesco De Logu, Romina Nassini, Alan Hegron, Lorenzo Landini, Dane D. Jensen, Rocco Latorre, Julia Ding, Matilde Marini, Daniel Souza Monteiro de Araujo, Paulina Ramírez-Garcia, Michael Whittaker, Jeffri Retamal, Mustafa Titiz, Alessandro Innocenti, Thomas P. Davis, Nicholas Veldhuis, Brian L. Schmidt, Nigel W. Bunnett, Pierangelo Geppetti

**Affiliations:** 1 Department of Health Sciences, Clinical Pharmacology and Oncology Section, University of Florence, Florence, 50139 Italy; 2 Headache Center, Careggi University Hospital, Florence, 50139 Italy; 3 Department of Molecular Pathobiology, College of Dentistry, New York University, New York, NY 10010 USA; 4 Bluestone Center for Clinical Research, New York University College of Dentistry, New York, NY 10010 USA; 5 Department of Anesthesiology, Columbia University, New York, NY 10010 USA; 6 Drug Discovery Biology Theme and Australian Research Council Centre of Excellence in Convergent Bio-Nano Science and Technology, Monash Institute of Pharmaceutical Sciences, Monash University, Parkville, VIC 3052 Australia; 7 Plastic and Reconstructive Microsurgery - Careggi University Hospital, Florence, 50139 Italy; 8 Australian Institute for Bioengineering and Nanotechnology, The University of Queensland, Brisbane, QLD 4072 Australia; 9 Department of Neuroscience and Physiology and Neuroscience Institute, School of Medicine, New York University, New York, NY 10010 USA

**Keywords:** G protein-coupled receptors, Endosomes, Schwann cell, Migraine

## Abstract

Efficacy of monoclonal antibodies against calcitonin gene-related peptide (CGRP) or its receptor (calcitonin receptor-like receptor/receptor activity modifying protein-1, CLR/RAMP1) implicates peripherally-released CGRP in migraine pain. However, the site and mechanism of CGRP-evoked peripheral pain remain unclear. By cell-selective RAMP1 gene deletion, we reveal that CGRP released from mouse cutaneous trigeminal fibers targets CLR/RAMP1 on surrounding Schwann cells to evoke periorbital mechanical allodynia. CLR/RAMP1 activation in human and mouse Schwann cells generates long-lasting signals from endosomes that evoke cAMP-dependent formation of NO. NO, by gating Schwann cell transient receptor potential ankyrin 1 (TRPA1), releases ROS, which in a feed-forward manner sustain allodynia via nociceptor TRPA1. When encapsulated into nanoparticles that release cargo in acidified endosomes, a CLR/RAMP1 antagonist provides superior inhibition of CGRP signaling and allodynia in mice. Our data suggest that the CGRP-mediated neuronal/Schwann cell pathway mediates allodynia associated with neurogenic inflammation, contributing to the algesic action of CGRP in mice.

## Abstract (web summary)

The mechanism of CGRP-evoked peripheral pain is unclear. Here, the authors show that the CGRP-mediated neuronal/Schwann cell pathway mediates allodynia associated with neurogenic inflammation, contributing to the algesic action of CGRP in mice.

## Introduction

For almost a century it has been known that cutaneous tissue injury elicits a local vascular response, referred to as neurogenic inflammation, that is associated to a wider area of increased sensitivity to mechanical stimuli^1^. A subset of C-fiber primary afferents, which mediate neurogenic inflammation, is the main source of the neuropeptides substance P (SP) and calcitonin gene-related peptide (CGRP)^2,3^. In rodents, noxious stimuli such as capsaicin, a pungent agonist of the transient receptor potential vanilloid 1 (TRPV1) channel^4^, evoke the peripheral release of CGRP which induces arteriolar vasodilatation^2^ and of SP which elicits plasma protein extravasation^5^, and produce sensory responses, which encompasses acute nociception and prolonged mechanical allodynia^6^. Capsaicin administration to the human skin elicits a similar pattern of responses, consisting of local cutaneous vasodilatation and focal and transient burning pain (min) associated with widespread, sustained mechanical hypersensitivity (hrs)^7^. While CGRP has been identified as the mediator of neurogenic vasodilatation in rodents^2^ and humans^8^, the cellular and molecular mechanisms underlying mechanical allodynia associated with neurogenic inflammation are unknown.

Mechanistic studies in animal models and humans have highlighted the role of CGRP in migraine pain^9^. Thus, small molecule antagonists of the CGRP receptor and monoclonal antibodies against CGRP or its receptor can relieve migraine pain^10^. The poor blood-brain barrier penetration of some small-molecule antagonists^11,12^ and of monoclonal antibodies^13,14^ suggests a peripheral contribution to CGRP-mediated migraine pain. However, little is known about the proalgesic actions of CGRP in the periphery. In mice, intraplantar injection of CGRP evokes mechanical allodynia^15^ and systemic CGRP causes facial grimace^16^. Periorbital CGRP injection, while failing to evoke spontaneous nociceptive behavior, produces sustained (~4 h) periorbital mechanical allodynia (PMA)^17^. CGRP released from trigeminal peripheral terminals mediates PMA in mice^18^ evoked by systemic (intraperitoneal) administration of the pro-headache agent glyceryl trinitrate (GTN)^19^. Facial cutaneous allodynia is one component of the migraine attack^20,21^. Although the process that initiates migraine pain may originate in the central nervous system (CNS)^22,23^, the cell type and signaling pathway by which CGRP acts in the periphery to cause pain are unknown.

The CGRP receptor is a heterodimer of calcitonin receptor-like receptor (CLR), a G protein-coupled receptor (GPCR), and receptor activity-modifying protein 1 (RAMP1), a single transmembrane domain CLR chaperone^24^. These two components coexist in cells that mediate the actions of CGRP, for example, vascular myocytes^2^. Satellite glial cells and Schwann cells express CLR/RAMP1 and are closely associated with peptidergic sensory neurons^25^. While the extracellular space between the soma of trigeminal neurons and satellite glial cells is not a recognized locus for neurotransmission, the varicosities of C-fibers and the ensheathing Schwann cells are sites where neuropeptides, including CGRP^26^, are normally released. Schwann cells from rat sciatic nerve respond to CGRP by increasing intracellular cAMP levels^27^ and CLR/RAMP1 are expressed by Schwann cells that wrap CGRP + ve terminals of rat nociceptors^25,28,29^. Schwann cells mediate mechanical allodynia in mouse models of neuropathic and cancer pain^30,31^. Cutaneous Schwann cells can also directly activate sensory nerves to promote mechanical nociception^32^. Although GPCRs are usually considered to signal principally from the plasma membrane, GPCR kinases and β-arrestins (βARRs) rapidly terminate this signaling. Persistent endosomal signaling of GPCRs, including CLR/RAMP1, underlies sustained neuronal activation and nociception in the CNS^33–35^.

Herein, we hypothesized that mechanical allodynia associated with neurogenic inflammation is mediated by CGRP which targets CLR/RAMP1 in Schwann cells ensheathing peripheral endings of nociceptors. By selective RAMP1 gene deletion in Schwann cells, we reveal that CGRP released from trigeminal terminals causes PMA by paracrine signaling to the surrounding Schwann cells. We also hypothesized that persistent CGRP/CLR/RAMP1 signaling from endosomes in Schwann cells underlies sustained PMA. By using inhibitors of clathrin- and dynamin-mediated endocytosis and stimulus-responsive nanoparticles designed to release CLR/RAMP1 antagonists in acidified endosomes, we found that CLR/RAMP1 endosomal signaling results in a cAMP-dependent release of nitric oxide (NO), which activates transient receptor potential ankyrin 1 (TRPA1), a proalgesic channel and sensor of oxidative stress^36^.

## Results

### CGRP evokes PMA by activating Schwann cell CLR/RAMP1

**Fig. 1:**
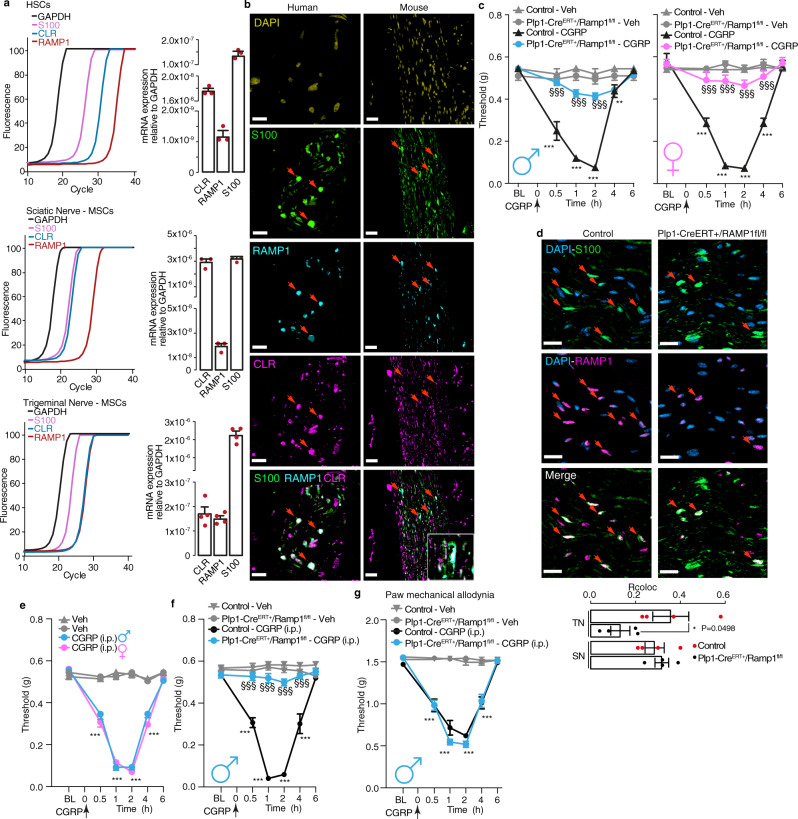
Schwann cell RAMP1 mediates PMA evoked by CGRP. **a** Representative real-time PCR plot and cumulative data for GAPDH, S100, CLR and RAMP1 mRNA in HSCs (*n* = 3 independent experiments) and MSCs from trigeminal or sciatic nerve (MSCs from sciatic nerve *n* = 3 independent experiments; MSCs from trigeminal nerve, *n* = 4 independent experiments). **b** Representative images of DAPI and immunoreactive S100, RAMP1 and CLR in human and mouse cutaneous nerve bundles (scale, 10 µm human, 50 μm mouse) (*n* = 3 subjects). **c** PMA induced by CGRP (1.5 nmol) or vehicle in male and female *Plp1-Cre*^*ERT+/*^*Ramp1*^*fl/fl*^ and Control mice treated with periorbital 4-OHT (*n* = 8 mice per group). **d** Representative images and colocalization value (Rcoloc) of S100 and RAMP1 in periorbital nerve and sciatic nerve trunks from *Plp1-Cre*^*ERT+*^*;Ramp1*^*fl/fl*^ and Control mice (scale, 20 μm) (*n* = 4 replicates). TN (trigeminal nerve), SN (sciatic nerve). **e** PMA induced by intraperitoneal (i.p.) CGRP (0.1 mg/kg) or vehicle in male and female C57BL/6 J mice (*n* = 8 mice per group). **f** PMA and (**g**) paw mechanical allodynia induced by intraperitoneal (i.p.) CGRP (0.1 mg/kg) or vehicle in male *Plp1-Cre*^*ERT+/*^*Ramp1*^*fl/fl*^ and Control mice (*n* = 8 mice per group) treated with periorbital 4-OHT. Mean±SEM. *P < 0.05, **P < 0.01, ***P < 0.001 vs. Veh, Control-Veh, and TN-Control, ^§§§^*P* < 0.001. vs. Control-CGRP. 2-way (**c**, **e**, **f**, **g**) or 1-way (d) ANOVA, Bonferroni correction. Source data are provided as a Source Data file.

We detected CLR and RAMP1 mRNA and immunoreactivity in primary cultures of human Schwann cells (HSCs) or mouse Schwann cells (MSCs) taken from the sciatic or trigeminal nerve (Fig. 1a, Supplementary Fig. 1a). The S100 + ve mouse Schwann cell line (IMS32) recapitulated features of primary MSCs, including expression of CLR and RAMP1 mRNA and immunoreactivity (Supplementary Fig. 1a, b) and TRPA1-dependent Ca^2+^ response to allyl isothiocyanate (Supplementary Fig. 1c). Immunoreactive CLR and RAMP1 were also detected in S100 + ve Schwann cells in nerve bundles in biopsies of human abdominal and mouse periorbital skin (Fig. 1b).

In C57BL/6 J male mice, periorbital CGRP elicited PMA (Supplementary Fig. 1d). Periorbital CGRP also evoked PMA in female mice (Supplementary Fig. 1d). Periorbital CGRP did not elicit allodynia in the hind paw (Supplementary Fig. 1e). Unless otherwise specified, all drugs were administered by subcutaneous periorbital injection (Supplementary Table 1). To assess the role of Schwann cell CLR/RAMP1 in CGRP-evoked PMA, Schwann cell-specific Cre mice (*Plp-Cre*^*ERT*^) were crossed with RAMP1 floxed mice *(Ramp1*^*fl/fl*^) to generate *Plp-Cre*^*ERT+*^*;Ramp1*^*fl/fl*^ and *Plp-Cre*^*ERT−*^*;Ramp1*^*fl/fl*^ (Control) mice. For selective deletion of RAMP1 in Schwann cells of the periorbital region, 4-hydroxytamoxifen (4-OHT) was administered daily for 3 days to *Plp-Cre*^*ERT+*^*;Ramp1*^*fl/fl*^ and Control mice. CGRP elicited PMA in both male and female Control mice that was similarly attenuated in *Plp-Cre*^*ERT+*^*;Ramp1*^*fl/fl*^ mice (Fig. 1c). In *Plp-Cre*^*ERT+*^*;Ramp1*^*fl/fl*^ mice 4-OHT treatment down-regulated RAMP1 immunoreactivity in S100 + ve cells surrounding trigeminal but not sciatic nerve fibers (Fig. 1d) and did not prevent paw mechanical allodynia evoked by intraplantar CGRP, which was prevented by intraplantar 4-OHT (Supplementary Fig. 1f).

Intravenous CGRP provokes delayed headache attacks in patients^37^. Intraperitoneal CGRP caused PMA and paw allodynia in male and female C57BL/6 J mice without gender difference (Fig. 1e, Supplementary Fig. 1g). In *Plp-Cre*^*ERT+*^*;Ramp1*^*fl/fl*^ mice treated with periorbital 4-OHT PMA, but not paw allodynia, was similarly reduced in males and females in response to intraperitoneal CGRP (Fig. 1f, g and Supplementary Fig. 1h, i). Systemic (intraperitoneal) 4-OHT reduced both PMA and paw allodynia by intraperitoneal CGRP (Supplementary Fig. 1j, k). These results reveal an essential role for CLR/RAMP1 of Schwann cells surrounding periorbital trigeminal endings in PMA elicited by local and systemic CGRP.

### Stimulation of peptidergic trigeminal neurons evokes PMA by activating Schwann cell CLR/RAMP1

**Fig. 2:**
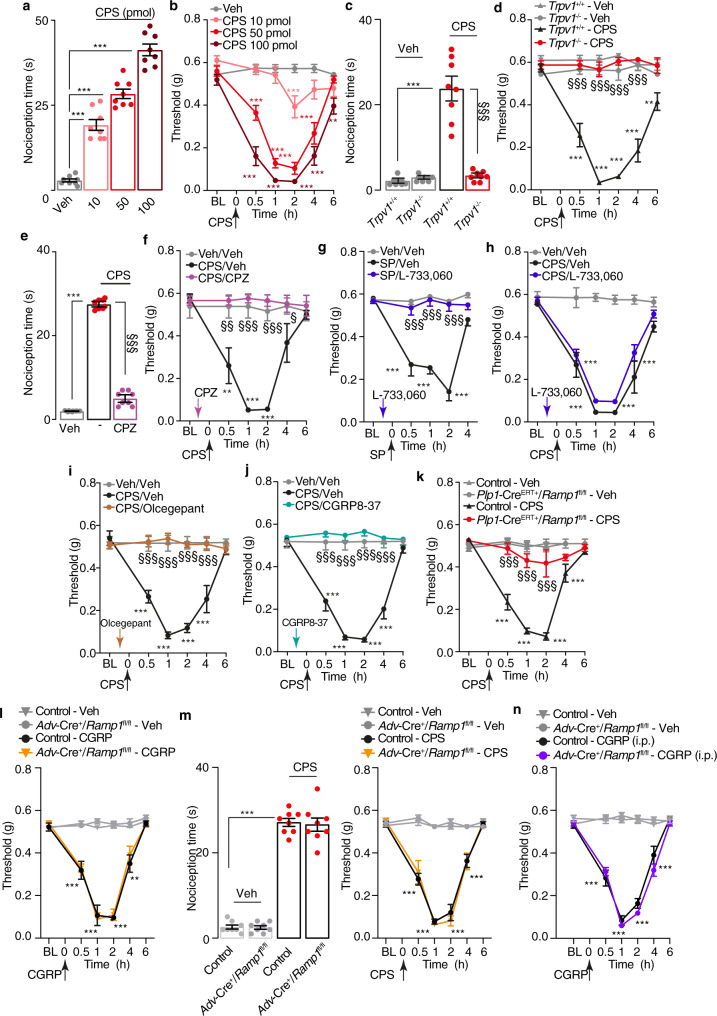
Capsaicin induces PMA via CGRP and CLR/RAMP1 in Schwann cells. **a** Acute nociception and (**b**) PMA after periorbital injection of capsaicin (CPS) or vehicle in C57BL/6 J mice. **c** Acute nociception and (**d**) PMA after CPS (50 pmol) or vehicle in *Trpv1*^*+/+*^ and *Trpv1*^*−/−*^ mice. **e** Acute nociception and (**f**) PMA after CPS (50 pmol) or vehicle in C57BL/6 J mice pretreated with capsazepine (CPZ, 100 pmol) or vehicle. **g**, **h** PMA after periorbital SP (3.5 nmol), CPS (50 pmol) or vehicle in C57BL/6 J mice pretreated (0.5 h) with L-733,060 (20 nmol) or vehicle. **i**, **j** PMA after CPS (50 pmol) or vehicle in C57BL/6 J mice pretreated (0.5 h) with olcegepant (1 nmol) or CGRP8-37 (10 nmol) or vehicle. **k** PMA after CPS (50 pmol) or vehicle in *Plp1-Cre*^*ERT+/*^*Ramp1*^*fl/fl*^ or Control mice treated with periorbital 4-OHT or vehicle. **l** PMA after periorbital CGRP (1.5 nmol) or vehicle and (**m**) acute nociceptive response and PMA after periorbital CPS (50 pmol) or vehicle in *Adv-Cre*^*+/*^*Ramp1*^*fl/fl*^ or Control mice. **n** PMA induced by intraperitoneal (i.p.) CGRP (0.1 mg/kg) or vehicle in *Adv-Cre*^*+/*^*Ramp1*^*fl/fl*^ or Control mice. Mean±SEM., *n* = 8 mice per group. **P < 0.01, ***P < 0.001 vs. Veh/Veh, Trpv1^+/+^-Veh and Control-Veh^; §^*P* < 0.05, ^§§^*P* < 0.01, ^§§§^*P* < 0.001 vs. Trpv1^+/+-^CPS, CPS/Veh, SP/Veh, Control-CPS, Control-CGRP. 1-way (**a**, **c**, **e** and **m** left panel) or 2-way (**b**, **d**, **f**–**l**, **m** right panel and **n**) ANOVA, Bonferroni correction. Source data are provided as a Source Data file.

To explore the ability of endogenous CGRP to elicit PMA, we administered capsaicin, which activates TRPV1^4^ thus releasing CGRP and SP from peptidergic nociceptors^3^. Periorbital capsaicin elicited acute (~10 min) nociceptive behavior (Fig. 2a) and, like CGRP, caused prolonged (~4 h) PMA (Fig. 2b). Allodynia was detected in the periorbital area but not in the hind paw (Supplementary Fig. 2a), indicating a local action. TRPV1 deletion (*Trpv1*^−/−^ mice) (Fig. 2c, d) or pretreatment with the TRPV1 antagonist capsazepine (Fig. 2e, f), prevented acute nociception and PMA. An antagonist (L-733,060) of the SP neurokinin 1 (NK_1_) receptor, which prevented SP-evoked PMA (Fig. 2g), failed to diminish capsaicin-evoked acute nociception and PMA (Fig. 2h, Supplementary Fig. 2b). The histamine H_1_ receptor antagonist, astemizole, inhibited SP-evoked PMA (Supplementary Fig. 2c) but did not affect CGRP- or capsaicin-evoked PMA (Supplementary Fig. 2d, e). Pretreatment with the CLR/RAMP1 antagonists, CGRP8-37 or olcegepant, prevented PMA (Fig. 2i, j) but not acute nociception evoked by capsaicin (Supplementary Fig. 2b). Importantly, 4-OHT markedly inhibited capsaicin-evoked PMA (Fig. 2k) but not acute nociception (Supplementary Fig. 2f) in *Plp-Cre*^*ERT+*^*;Ramp1*^*fl/fl*^ mice observed in Control mice. CGRP-evoked PMA or capsaicin-evoked acute nociception and PMA were similar in mice with selective deletion of RAMP1 in nociceptors (*Advillin-Cre*^*+*^*;Ramp1*^*fl/fl*^*, Adv-Cre*^*+*^*;Ramp1*^*fl/fl*^) and Control mice (Fig. 2l, m). Both PMA and paw allodynia were similar in *Adv-Cre*^*+*^*;Ramp1*^*fl/fl*^ and Control mice after intraperitoneal CGRP (Fig. 2n and Supplementary Fig. 3a). Thus, CGRP but not SP mediates allodynia resulting from excitation of TRPV1 + ve peptidergic nociceptors, and PMA evoked by both endogenous and exogenous CGRP depends on CLR/RAMP1 of Schwann cells surrounding peripheral terminals of nociceptors, while the receptor of sensory nerve fibers is not implicated.

### Schwann cell CLR/RAMP1 mediates the CGRP-dependent PMA evoked by GTN

**Fig. 3:**
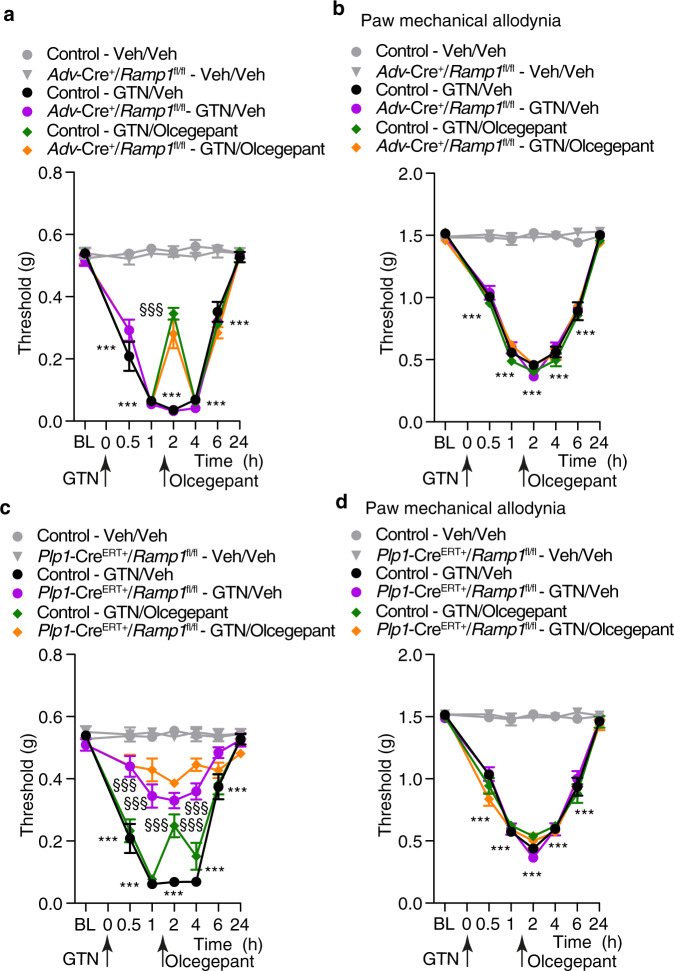
GTN induces PMA via CGRP released from periorbital trigeminal terminals and, CLR/RAMP1 in Schwann cells. **a** PMA and (**b**) paw mechanical allodynia induced by intraperitoneal (i.p.) GTN (10 mg/kg) or vehicle in *Adv-Cre*^*+/*^*Ramp1*^*fl/fl*^ or Control mice post-treated (1.5 hs after GTN) with olcegepant (1 nmol) or vehicle. (**c**) PMA and (**d**) paw mechanical allodynia induced by GTN (10 mg/kg, i.p.) or vehicle in *Plp1-Cre*^*ERT+/*^*Ramp1*^*fl/fl*^ or Control (treated with periorbital 4-OHT or vehicle) post-treated (1.5 hs after GTN) with olcegepant (1 nmol) or vehicle. Mean±SEM., *n* = 8 mice per group. ***P < 0.001 vs. Control-Veh/Veh; ^§§§^*P* < 0.001 vs. Control-GTN/Veh. 2-way ANOVA, Bonferroni correction. Source data are provided as a Source Data file.

Systemic GTN administration provokes sustained headaches in humans^19^. In mice intraperitoneal GTN elicits PMA^18^ that is, in part mediated by CGRP release from periorbital trigeminal terminals^18^. Here, systemic GTN elicited PMA (Fig. 3a) and paw allodynia (Fig. 3b) that were similar in Control and *Adv-Cre*^*+*^*;Ramp1*^*fl/fl*^ mice. Olcegepant transiently and partially inhibited PMA (Fig. 3a), while did not affect GTN-evoked paw allodynia (Fig. 3b) in both mouse strains. RAMP1 deletion from trigeminal Schwann cells (*Plp-Cre*^*ERT+*^*;Ramp1*^*fl/fl*^ mice) partially inhibited PMA (Fig. 3c), but not paw allodynia (Fig. 3d). Importantly, treatment with olcegepant reduced GTN-evoked PMA in Control mice, but failed to further inhibit the response in *Plp-Cre*^*ERT+*^*;Ramp1*^*fl/fl*^ mice (Fig. 3c). Paw allodynia was unchanged by olcegepant (Fig. 3d). Together, the findings suggest that Schwann cell CLR/RAMP1 mediates the CGRP-dependent component of PMA in a mouse headache model.

### Clathrin- and dynamin-mediated endocytosis of CLR/RAMP1 in Schwann cells mediates PMA

**Fig. 4:**
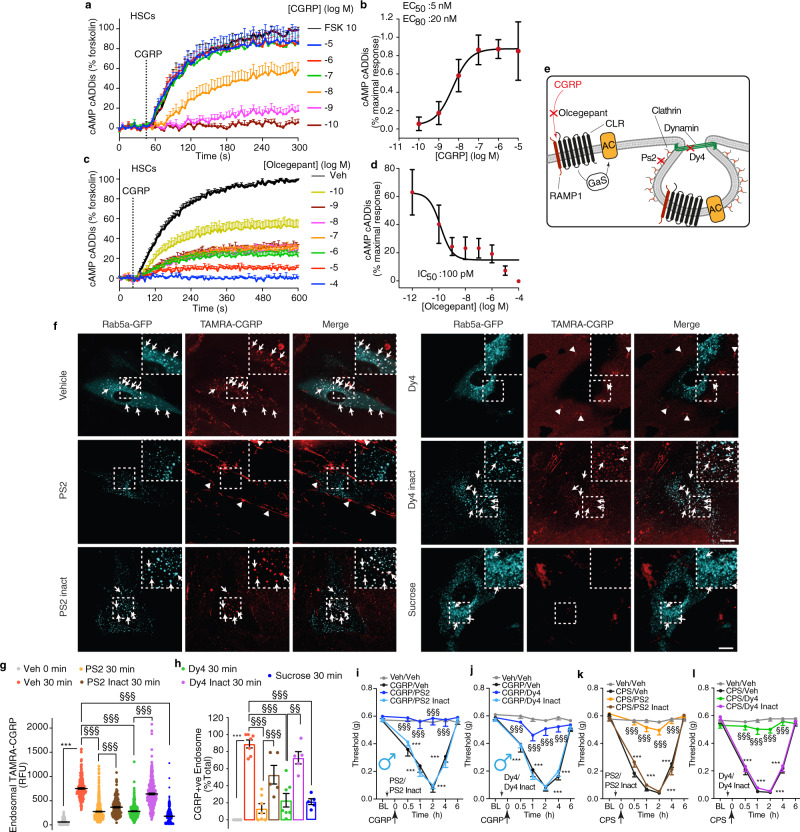
Functional CLR/RAMP1 is expressed by HSCs and undergoes clathrin- and dynamin-mediated endocytosis, which underlies nociception. **a**, **b** Effects of graded concentrations of CGRP on cAMP formation (*n* = 5 independent experiments). **c**, **d** Effects of graded concentrations of olcegepant on CGRP (100 nM)-evoked cAMP formation (*n* = 4 independent experiments). **e** Pharmacological targets. **f** Representative images of HSCs expressing Rab5a-GFP at 30 min after incubation with TAMRA-CGRP (100 nM). Arrows denote colocalization of TAMRA-CGRP and Rab5a-GFP. Arrowheads denote retention of a weak TAMRA-CGRP signal at the plasma membrane. Cells were preincubated with vehicle, Dyngo-4a (Dy4), Pitstop 2 (PS2), inactive analogs (PS2 and Dy4 inact) (all 30 µM) or sucrose (0.45 M) (*n* = 4 independent experiments). Scale, 10 µm. **g** Quantification of localization of TAMRA-CGRP in endosomes (data represent *n* = 949 veh 0 min, *n* = 1209 veh 30 min, *n* = 1111 Dy4 30 min, *n* = 1016 Dy4 inact 30 min, *n* = 1700 PS2 30 min, *n* = 714 PS2 inact 30 min, *n* = 1896 sucrose) and (**h**) quantification of the number of TAMRA-CGRP+ve endosomes (data represent *n* = 5 veh 0 min, *n* = 7 veh 30 min, *n* = 7 Dy4 30 min, *n* = 5 Dy4 inact 30 min, *n* = 7 PS2 30 min, *n* = 5 PS2 inact 30 min, *n* = 5 sucrose). **i**, **j** PMA induced by periorbital CGRP (1.5 nmol) or vehicle in C57BL/6 J male mice pretreated (0.5 h) with PS2, Dy4, PS2 or Dy4 inact (all 500 pmol) (*n* = 8 mice per group). **k**, **l** PMA induced by periorbital capsaicin (CPS, 50 pmol) or vehicle in C57BL/6 J male mice pretreated (0.5 h) with PS2, Dy4, PS2 or Dy4 inact (all 500 pmol) (*n* = 8 mice per group). Mean±SEM. ***P < 0.001 vs. Veh 0 min, and Veh/Veh; ^§§^*P* < 0.01, ^§§§^*P* < 0.001 vs. Veh 30 min, PS2 30 min, Dy4 30 min, CGRP/PS2 inact, CGRP/Dy4 inact, CPS/PS2 inact, CPS/Dy4 inact. 1-way (**g**, **h**) or 2-way (**i**–**l**) ANOVA, Bonferroni correction. Source data are provided as a Source Data file.

We investigated the CLR/RAMP1 signaling pathway in Schwann cells that mediates CGRP-evoked PMA. The CGRP-stimulated cAMP formation was measured in HSCs using a virally encoded cAMP cADDis reporter. CGRP stimulated a prompt concentration-dependent increase in cAMP formation in HSCs that was sustained for >300 s (Fig. 4a, b; Supplementary Movie 1). The CLR/RAMP1 antagonist olcegepant caused a concentration-dependent inhibition of CGRP-stimulated cAMP formation (Fig. 4c–e).

Endosomal signaling of GPCRs, including CLR/RAMP1, controls nociception^33–35^. To assess CLR/RAMP1 endocytosis in Schwann cells, we incubated HSCs expressing the early endosome marker Rab5a-GFP with TAMRA-CGRP. In vehicle-treated cells, live-cell imaging revealed uptake of TAMRA-CGRP into Rab5a-GFP + ve early endosomes within 10 min that continued for 30 min (Fig. 4f, Supplementary Movie 2). Inhibitors of clathrin (PitStop2, PS2) or dynamin (Dyngo4a, Dy4) prevented the translocation of TAMRA-CGRP to endosomes causing retention of weak TAMRA-CGRP fluorescence at the cell surface (Fig. 4e, f; Supplementary Movies 3 and 4). Quantification of TAMRA-CGRP fluorescence intensity in Rab5a-GFP + ve endosomes or the proportion of endosomes containing TAMRA-CGRP confirmed that PS2 and Dy4 inhibited endocytosis of TAMRA-CGRP (Fig. 4g, h). Inactive analogs had no effect. Hypertonic sucrose (0.45 M) inhibits clathrin-mediated endocytosis, including agonist-stimulated endocytosis of GPCRs^38^. Hypertonic sucrose also inhibited the uptake of TAMRA-CGRP in HSCs (Fig. 4g, h, Supplementary Movie 5). Injection of PS2 or Dy4, but not their inactive analogs, prevented CGRP-evoked PMA, both in male and female mice (Fig. 4i, j and Supplementary Fig. 3b, c). PS2 or Dy4 also reversed capsaicin-evoked PMA (Fig. 4k, l). Thus, CGRP stimulates clathrin- and dynamin-mediated endocytosis of CLR/RAMP1 in Schwann cells, which sustains CGRP-evoked PMA.

### CLR activates Gα_s_, Gα_q_, and Gα_i_ and recruits βARR2 to the plasma membrane and endosomes

**Fig. 5:**
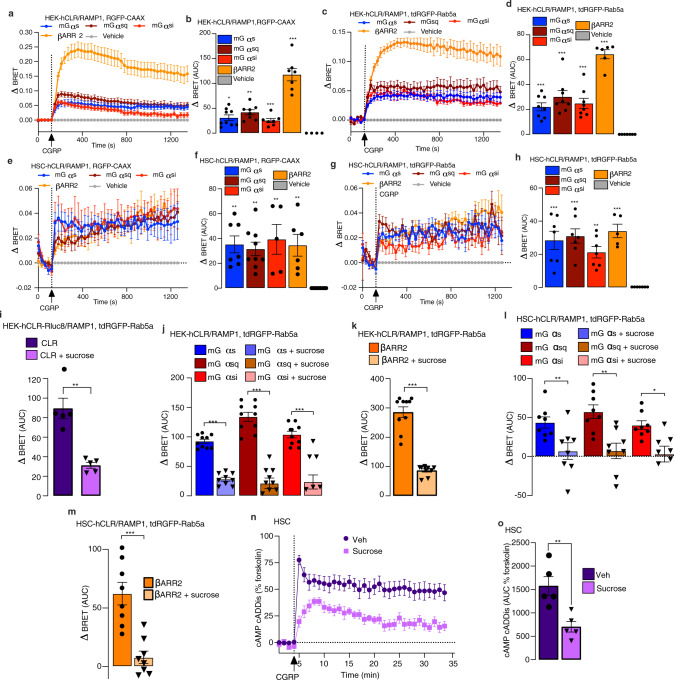
CGRP leads to Gα protein activation and βARR2 recruitment at the plasma membrane and in early endosomes in HEK-hCLR/RAMP1 cells and HSCs-hCLR/RAMP1. Endosomal signaling generates sustained formation of cAMP in HSCs. (**a**–**d**) CGRP (100 nM) increased EbBRET between Rluc8-mGα_s_, Rluc8-mGα_sq_, Rluc8-mGα_si_, and Rluc2-βARR2 with RGFP-CAAX (**a**, **b**) and tdRGFP-Rab5a (**c**, **d**) in HEK-hCLR/RAMP1 cells. **a**, **c** time course. **b**, **d** area under curve (AUC) (**a**, **b** *n* = 10 mGα_s_, *n* = 8 mGα_sq,_ *n* = 7 mGα_si,_ *n* = 7 βARR2, *n* = 9 veh; **c**, **d** *n* = 8 mGα_s_, *n* = 8 mGα_sq,_ *n* = 8 mGα_si,_ *n* = 6 βARR2, *n* = 9 veh). **e**–**h** CGRP (100 nM) increased EbBRET between Rluc8-mGα_s_, Rluc8-mGα_sq_, Rluc8-mGα_si_, and Rluc2-βARR2 with RGFP-CAAX (**e**, **f**) and tdRGFP-Rab5a (**g**, **h**) in HSC-hCLR/RAMP1 cells. **e**, **g** time course. **f**, **h** AUC (**e**, **f** *n* = 7 mGα_s_, *n* = 9 mGα_sq,_ *n* = 5 mGα_si,_ *n* = 6 βARR2, *n* = 9 veh; **g**, **h** *n* = 7 mGα_s_, *n* = 7 mGα_sq,_ *n* = 7 mGα_si,_ *n* = 5 βARR2, *n* = 7 veh). (**i**) Hypertonic sucrose (0.45 M) inhibited CGRP (100 nM)-stimulated EbBRET between hCLR-Rluc8 and tdRGFP-Rab5a in HEK-hCLR/RAMP1 cells (*n* = 5 independent experiments). (**j**, **k**) Hypertonic sucrose (0.45 M) inhibited CGRP (100 nM)-stimulated EbBRET between Rluc8-mGα_s_, Rluc8-mGα_sq_, Rluc8-mGα_si_ and Rluc2-βARR2 with tdRGFP-Rab5a in HEK-hCLR/RAMP1 cells (**j**, **k** *n* = 10 independent experiments). (**l**, **m**) Sucrose (0.45 M) inhibited CGRP (100 nM)-stimulated EbBRET between Rluc8-mGα_s_, Rluc8-mGα_sq_, Rluc8-mGα_si_, and Rluc2-βARR2 with tdRGFP-Rab5a in HSC-hCLR/RAMP1 cells. (*n* = 8 independent experiments). (**n**, **o**) Sucrose (0.45 M) inhibited CGRP (100 nM)-stimulated formation of cAMP in HSCs. **n** time course. o, AUC (*n* = 5 independent experiments). Mean±SEM.. *P < 0.05, **P < 0.01, ***P < 0.001, vs. Veh. 1-way ANOVA, Dunnett’s correction (**b**, **d**, **f**, **h**) or parametric unpaired *t* test (**i**–**m**, **o**). Source data are provided as a Source Data file.

GPCRs, including CLR/RAMP1, can signal from endosomes by Gα_s_, Gα_q_, and βARR-mediated mechanisms^33–35^. We used enhanced bystander bioluminescence resonance energy transfer (EbBRET) to study the activation of Gα and recruitment of βARR to the plasma membrane and early endosomes of HEK293T cells expressing human (h) CLR and RAMP1 (HEK-hCLR/RAMP1). CGRP-dependent activation of Gα_s_, Gα_sq_, and Gα_si_ was assessed using an EbBRET assay that detects recruitment of mini (m) Gα coupled to *Renilla* (R)luc8 to the plasma membrane marker CAAX coupled to RGFP or the early endosome marker Rab5a coupled to tandem (td)RGFP. mGα proteins are N-terminally truncated Gα proteins that freely diffuse throughout the cytoplasm and bind to active conformations of GPCRs. Their translocation to GPCRs reflects Gα activation. mGα_sq_ and mGα_si_ were developed by mutating mGα_s_ residues to equivalent Gα_q_ and Gα_i_ residues. Recruitment of βARR was assessed by measuring EbBRET between Rluc2-βARR2 and RGFP-CAAX or tdRGFP-Rab5a. CGRP induced a rapid increase in EbBRET between Rluc8-mGα_s_, Rluc8-mGα_sq_, Rluc8-mGα_si_, and Rluc2-βARR2 with RGFP-CAAX, which was maximal at ~300 s and declined over 1000 s (Fig. 5a, b). CGRP increased EbBRET between Rluc8-mGα_s_, Rluc8-mGα_sq_, Rluc8-mGα_si_ and Rluc2-βARR2 with tdRGFP-Rab5a that was fully sustained for 1300 s (Fig. 5c, d). EbBRET was similarly used to study the activation of Gα and recruitment of βARR2 to the plasma membrane and endosomes of HSCs transfected with hCLR/RAMP1 (CLR/RAMP1 overexpression was required to amplify BRET signals). In HSCs, CGRP increased EbBRET between Rluc8-mGα_s_, Rluc8-mGα_sq_, Rluc8-mGα_si_ and Rluc2-βARR2 with RGFP-CAAX and tdRGFP-Rab5a (Fig. 5e–h). EbBRET signals were sustained for 1300 s.

To investigate the contribution of endocytosis to the activation of Gα proteins and βARRs in endosomes, we preincubated cells with hypertonic sucrose. CGRP increased EbBRET between hCLR-Rluc8 and tdRGFP-Rab5a in HEK-hCLR/RAMP1 cells, consistent with CLR endocytosis (Fig. 5i). Hypertonic sucrose inhibited these changes, which indicates an inhibition of endocytosis (Fig. 5i). Hypertonic sucrose caused a delayed yet more sustained activation of Rluc8-mGα_s_, Rluc8-mGα_sq_, Rluc8-mGα_si_, and Rluc2-βARR2 at the plasma membrane (Supplementary Fig. 4a–f), and an almost complete inhibition of activation of Rluc8-mGα_s_, Rluc8-mGα_sq_, Rluc8-mGα_si_ and Rluc2-βARR2 in endosomes (Fig. 5j, k). Sucrose similarly delayed CGRP-induced recruitment of Rluc8-mGα_s_, Rluc8-mGα_sq_, Rluc8-mGα_si_ and Rluc2-βARR2 to the plasma membrane (Supplementary Fig. 4g–l) and almost completely inhibited activation of Gα_s,sq,si_ and βARR2 in endosomes of HSCs expressing hCLR/RAMP1 (Fig. 5l, m).

To examine the contribution of endosomal CLR/RAMP1 signaling to CGRP-induced cAMP formation, we preincubated HSCs expressing the cADDis cAMP reporter with sucrose or vehicle. In vehicle-treated cells, CGRP stimulated a rapid (1 min) increase in cAMP formation that was sustained for 30 min (Fig. 5n, o). Sucrose reduced but did not abolish the initial response, yet strongly inhibited the sustained phase of CGRP-stimulated cAMP formation (Fig. 5n, o). Thus, CGRP initially activates Gα and βARR at the plasma membrane, which is followed by sustained activation of Gα and βARR in early endosomes. Endocytosis is necessary for the recruitment of Gα and βARR to endosomes. Gα_s_ continues to signal in endosomes, leading to sustained cAMP formation.

### CLR/RAMP1 activation in Schwann cells releases NO, which initiates but does not sustain PMA

**Fig. 6:**
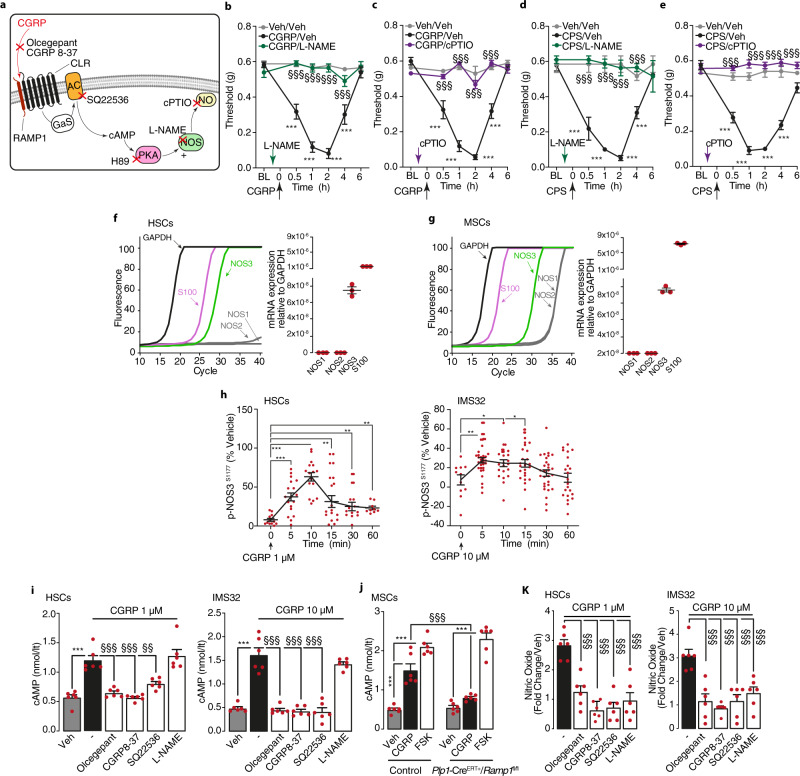
Endogenous and exogenous CGRP induces PMA via NO production. **a** Pharmacological targets. **b**–**e** PMA after periorbital injection of CGRP (1.5 nmol), capsaicin (CPS, 50 pmol) or vehicle in C57BL/6 J mice pretreated (0.5 h) with L-NAME (1 μmol) and cPTIO (200 nmol) or vehicle (*n* = 8 mice per group). **f**, **g** Real-time PCR plot and cumulative data for GAPDH, S100, NOS1, NOS2 and NOS3 in primary HSCs and MSCs (*n* = 3 independent experiments). **h** In-cell p-NOS3^s1177^ ELISA in HSCs and IMS32 cells before (time 0, *n* = 11 independent experiments in both HSCs and IMS32) or after CGRP (time 5, *n* = 18 and *n* = 31, time 10, *n* = 16 and *n* = 21, time 15, *n* = 15 and *n* = 25, time 30, *n* = 12 and *n* = 29, time 60, *n* = 9 and *n* = 22 independent experiments in HSCs and IMS32, respectively). cAMP assay in (**i**) HSCs, IMS32 and (**j**) MSCs from *Plp1-Cre*^*ERT+/*^*Ramp1*^*fl/fl*^ or Control mice treated with intraperitoneal 4-OHT, **k** nitric oxide assay in HSCs and IMS32 cells treated with CGRP or vehicle (*n* = 6 independent experiments). Some cells were treated with olcegepant (1 µM), CGRP8-37 (1 µM), SQ22536 (100 µM), L-NAME (100 µM) or vehicle (*n* = 6 independent experiments). Mean±SEM. (−) represents the combination of different vehicles. *P < 0.05, **P < 0.01, ***P < 0.001 vs. Veh/Veh, time 0 (min); ^§§§^*P* < 0.001 vs. CGRP/Veh, CGRP 1 µM and 10 µM. 1-way (**h**–**k**) or 2-way (**b**–**e**) ANOVA, Bonferroni correction. Source data are provided as a Source Data file.

We investigated the mechanisms that sustain PMA following CLR/RAMP1 activation and endocytosis in Schwann cells. Pre- but not post-treatment (60 min after CGRP or capsaicin) with CLR/RAMP1 antagonists, olcegepant or CGRP8-37, attenuated PMA evoked by capsaicin and in accordance with previous studies^17,18^ PMA evoked by CGRP (Supplementary Fig. 5a–f). Similarly, inhibitors of clathrin- and dynamin-mediated endocytosis had no effect when administered 60 min after CGRP or capsaicin (Supplementary Fig. 5g–j). Thus, once induced by CGRP, CLR/RAMP1 antagonists or inhibitors of CRL/RAMP1 internalization are unable to attenuate PMA. Pre- but not post-treatment with the protein kinase A (PKA) inhibitor, H89, reduced PMA by CGRP and capsaicin (Supplementary Fig. 5k–n). NO has been implicated in CGRP-mediated vascular responses^2^. Although NO can release CGRP with proalgesic functions, the contribution of NO to CGRP-evoked allodynia is uncertain. Pretreatment with an NO synthase (NOS) inhibitor (L-NAME) or an NO scavenger (cPTIO) (Fig. 6a), abrogated CGRP-evoked PMA (Fig. 6b, c). L-NAME and cPTIO pretreatment also attenuated capsaicin-evoked PMA (Fig. 6d, e). However, L-NAME and cPTIO did not affect PMA when administered 60 min after CGRP (Supplementary Fig. 5o, p) or capsaicin (Supplementary Fig. 5q, r). Thus, PKA-dependent NO release^39^ is necessary to initiate, but is not sufficient to sustain, CGRP-evoked allodynia.

In vitro findings recapitulated in vivo results. HSCs, MSCs, and IMS32 cells predominantly expressed NOS3 (eNOS) mRNA, with little or no expression of NOS1 and NOS2 (nNOS and iNOS, respectively) mRNA (Fig. 6f, g; Supplementary Fig. 5s). In both HSCs and IMS32 cells CGRP elicited a transient increase in NOS3 phosphorylation (*i.e*., activation), consistent with NO generation, which peaked at 5–10 min and declined within 30–60 min (Fig. 6h), and a cAMP increase that was prevented by olcegepant, CGRP8-37 and an adenylyl cyclase inhibitor (SQ22536), but not by L-NAME (Fig. 6i). The increase in cAMP evoked by CGRP but not that elicited by forskolin was reduced in cultured MSCs obtained from *Plp-Cre*^*ERT+*^*;Ramp1*^*fl/fl*^ mice as compared to Control mice treated with intraperitoneal 4-OHT (Fig. 6j). In contrast, the CGRP-evoked increase in NO was attenuated by all these interventions, including NOS inhibition (Fig. 6k). The cAMP increase evoked by forskolin was unaffected by CLR/RAMP1 antagonism and NOS inhibition, and olcegepant failed to inhibit NO release by the NO donor NONOate (Supplementary Fig. 5t, u), indicating selectivity. NO release evoked by CGRP, but not that evoked by NONOate was inhibited by PS2 and Dy4, but not their inactive analogs (Supplementary Fig. 5v), further supporting selectivity. These results suggest that clathrin- and dynamin-dependent endocytosis and endosomal CLR/RAMP1 signaling evoke NOS activation and NO generation in Schwann cells.

### Schwann cell TRPA1 mediates CGRP-evoked PMA

**Fig. 7:**
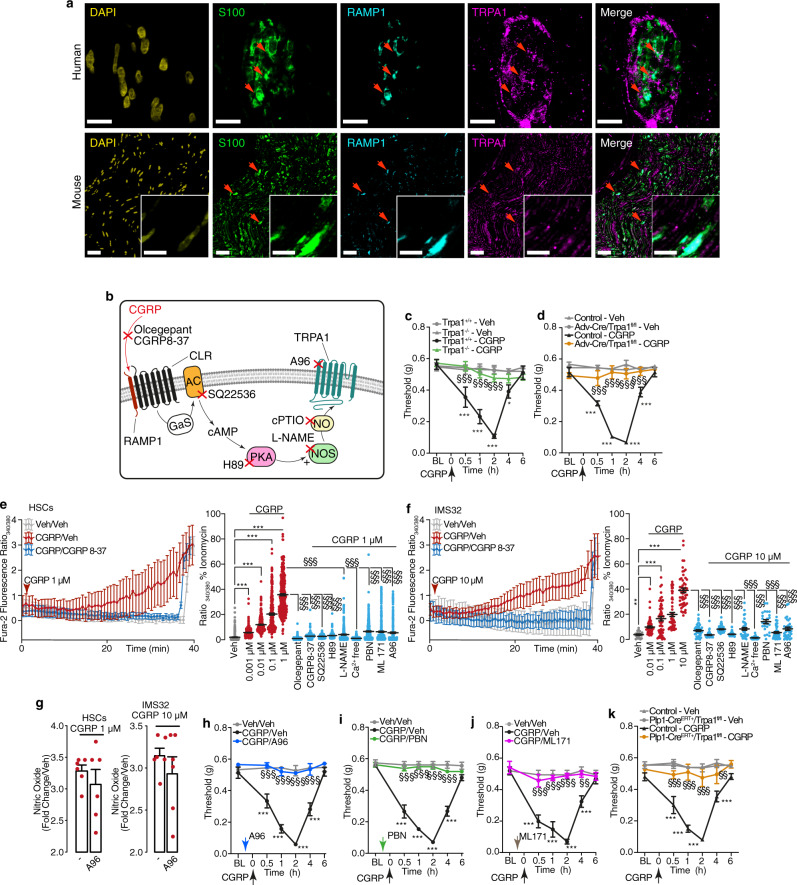
CGRP induces ROS release via Schwann cell TRPA1 activation. **a** Representative images of localization of immunoreactive DAPI, S100, RAMP1 and TRPA1 in human abdominal and mouse periorbital cutaneous nerve bundles (Scale: 10 µm human, 50 μm mouse, inset 10 µm) (*n* = 3 subjects). **b** Pharmacological targets. **c**, **d** PMA after periorbital injection of CGRP (1.5 nmol) or vehicle in (**c**) *Trpa1*^*+/+*^ and *Trpa1*^−/−^ mice and in (**d**) *Adv-Cre*^*+*^*/Trpa1*^*fl/fl*^ or Control mice (*n* = 8 mice per group). **e**, **f** Ca^2+^ response in HSCs and IMS32 cells exposed to CGRP (0.001 µM, HSCs *n* = 284; 0.01 µM, HSCs *n* = 291, IMS32 *n* = 62; 0.1 µM, HSCs *n* = 297, IMS32 *n* = 53; 1 µM, HSCs *n* = 362, IMS32 *n* = 57; 10 µM, IMS32 *n* = 53 cells) in the presence of olcegepant (100 nM, HSCs *n* = 291, IMS32 *n* = 44 cells), CGRP8-37 (100 nM, HSCs *n* = 359, IMS32 *n* = 55 cells), SQ22536 (100 μM, HSCs *n* = 225, IMS32 *n* = 47 cells), H89 (1 µM, HSCs *n* = 292, IMS32 *n* = 58 cells), L-NAME (10 μM HSCs *n* = 285, IMS32 *n* = 38), Ca^2+^-free medium (HSCs *n* = 290, IMS32 *n* = 28 cells), PBN (50 μM, HSCs *n* = 309, IMS32 *n* = 24 cells), ML171 (1 μM, HSCs *n* = 320, IMS32 *n* = 49 cells), A967079 (A96, 50 μM, HSC*s n* = 276 IMS32 *n* = 533 cells) or vehicle (HSCs *n* = 297, IMS32 *n* = 41 cells). **g** Nitric oxide release in HSCs and IMS32 cells exposed to CGRP (1 or 10 µM) in the presence of A96 (50 μM) or vehicle (n = 6 independent experiments). (**h**–**j**) PMA after CGRP (1.5 nmol) or vehicle in C57BL/6 J male mice pre-treated (0.5 h) with **h** A96 (300 nmol), **i** PBN (670 nmol) or (**j**) ML171 (250 nmol) or vehicle. **k** PMA after CGRP (1.5 nmol) or vehicle in *Plp1-Cre*^*ERT+/*^*Trpa1*^*fl/fl*^ or Control mice treated with 4-OHT (**h**–**k** *n* = 8 mice per group). Mean±SEM. (−) represents the combination of different vehicles. *P < 0.05, **P < 0.01, ***P < 0.001 vs. *Trpa1*^*+/+*^*-*Veh, Control-Veh, Veh and Veh/Veh*;* ^§§^*P* < 0.01, ^§§§^*P* < 0.001 vs. *Trpa1*^*+/+-*^CGRP, Control-CGRP, CGRP 1 µM and 1 µM, CGRP/Veh. 2-way (**c**, **d**, **h**-**k**) or 1-way (**e**, **f**) ANOVA, Bonferroni correction. Source data are provided as a Source Data file.

NO belongs to a series of reactive oxygen species (ROS) that target TRPA1^40^. TRPA1 is coexpressed with TRPV1 and CGRP in a subpopulation of primary sensory neurons^41^. TRPA1 is expressed in Schwann cells of nerve bundles of human skin and mouse sciatic nerve, where it mediates mechanical allodynia in rodent models of pain^30,42^. Immunoreactive TRPA1 was coexpressed with RAMP1 in S100 + ve Schwann cells in human abdominal and mouse periorbital cutaneous nerves bundles (Fig. 7a). Thus, CLR/RAMP1 might engage signaling pathways that activate TRPA1 in trigeminal Schwann cells to initiate allodynia (Fig. 7b). This hypothesis was supported by the observation that both CGRP- and capsaicin-evoked PMA were reduced in *Trpa1*^−/−^ mice and in mice with sensory neuron-specific deletion of TRPA1 (*Adv-Cre*^*+*^*;Trpa1*^*fl/fl*^) (Fig. 7c, d and Supplementary Fig. 6a, b).

We next investigated the signaling pathway by which the CLR/RAMP1 activates TRPA1. In HSCs and IMS32 cells, CGRP stimulated a slowly developing yet sustained increase in Ca^2+^ response (Fig. 7e, f) and increased H_2_O_2_ levels (Supplementary Fig. 6c). Olcegepant, CGRP8-37, SQ22536, H89, L-NAME, Ca^2+^-free medium, a ROS scavenger (PBN) or a NOX1 inhibitor (ML171) attenuated Ca^2+^ responses (Fig. 7e, f) and H_2_O_2_ levels (Supplementary Fig. 6c). A TRPA1 antagonist (A967079) inhibited CGRP-stimulated Ca^2+^ (Fig. 7e, f) and H_2_O_2_ responses (Supplementary Fig. 6c) but did not affect CGRP-stimulated NO formation (Fig. 7g). CGRP-evoked Ca^2+^ responses were reduced in Schwann cells from *Trpa1*^−/−^ mice (Supplementary Fig. 6d). These results support the hypothesis that CGRP liberates NO, which activates Schwann cell TRPA1; activated TRPA1 promotes a Ca^2+^-dependent H_2_O_2_ generation that sustains a feed-forward mechanism comprising TRPA1 channel engagement and ROS release.

In vivo results corroborated this hypothesis. Whereas CLR/RAMP1 antagonists or NO inhibitors attenuated PMA only if given before CGRP or capsaicin, both pre- and post-treatment with a TRPA1 antagonist, a ROS scavenger and a NOX1 inhibitor reduced PMA (Fig. 7h–j; Supplementary Fig. 6e–g). Although pretreatment with TRPA1 or ROS inhibitors did not affect the acute nociception, they inhibited capsaicin-evoked PMA (Supplementary Fig. 6h–j). Post-treatment also attenuated capsaicin-evoked PMA (Supplementary Fig. 6h–j). These findings highlight the mechanistic differences between acute nociception and delayed PMA. After an initial and transient NO-dependent phase, PMA is sustained by persistent ROS liberation, which targets TRPA1 in Schwann cells. This hypothesis is robustly supported by the observation that PMA evoked by CGRP or capsaicin was markedly attenuated in mice with selective deletion of TRPA1 in Schwann cells (*Plp-Cre*^*ERT+*^*;Trpa1*^*fl/fl*^) (Fig. 7k; Supplementary Fig. 6k).

### Targeting endosomal CGRP signaling provides superior relief of CGRP- and capsaicin-evoked PMA

The finding that persistent GPCR signaling from endosomes mediates pain transmission suggests that GPCRs in endosomes rather than at the plasma membrane are a valid and perhaps superior target for the treatment of pain^33–35^. Nanoparticles have been used to deliver chemotherapeutics to tumor, where endocytosis and endosomal escape are necessary for drug delivery to cytosolic and nuclear targets^43^. The realization that GPCRs within endosomes are a therapeutic target, raises the possibility of exploiting the acid microenvironment of endosomes as a stimulus for nanoparticle disassembly and release of antagonist cargo^34^.

**Fig. 8:**
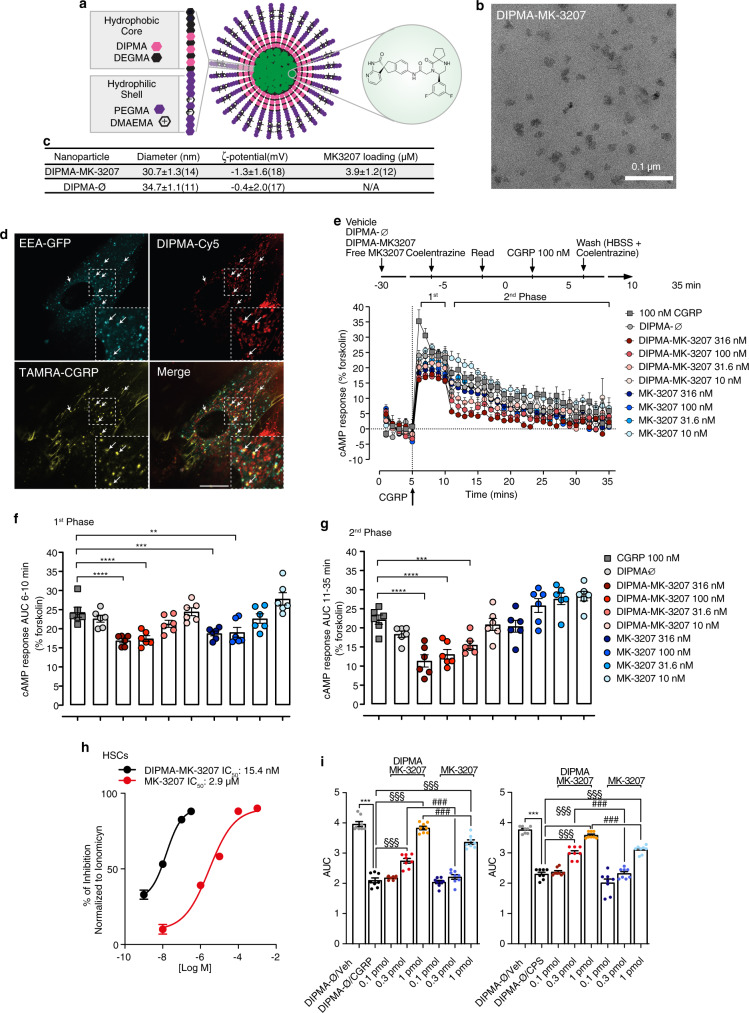
DIPMA-MK-3207 nanoparticles target endosomal CLR/RAMP1 signaling and provide superior relief from CGRP-evoked PMA. **a** pH-responsive DIPMA-MK-3207. **b** Transmission electron micrograph image of DIPMA-MK-3207 (Scale: 0.1 µm), from two different nanoparticle preparations (3 images captured per sample). **c** Physicochemical properties of DIPMA-MK-3207 and DIPMA-Ø. **d** Uptake of DIPMA-Cy5 into HSCs expressing EEA1-GFP. Cells were preincubated with DIPMA-Cy5 (40–60 ng/ml) for 30 min and were then incubated with TAMRA-CGRP (100 nM) for 30 min. Arrows denote accumulation of TAMRA-CGRP in early endosomes containing DIPMA-Cy5. Representative images from *n* = 5 independent experiments (Scale: 10 µm). **e**–**g** Effects of DIPMA-MK-3207, MK-3207, DIPMA-Ø or vehicle on CGRP- (100 nM) stimulated cAMP formation in HEK-rCLR/RAMP1 cells. **e** Time course and **f**, **g** integrated response (AUC) before (1st phase) and after (2nd phase) washing to remove extracellular CGRP (*n* = 6 independent experiments). **h** Concentration-response curves of the inhibition by DIPMA-MK-3207 or free MK-3207 on the Ca^2+^ response to CGRP in HSCs (DIPMA-MK-3207: −9M, *n* = 145; −8M, *n* = 361; −7M, *n* = 213: −6.5 M, *n* = 150; or free MK-3207: −8M, *n* = 83; −6M, *n* = 106; −5M, *n* = 87; −4M, *n* = 127: −3M, *n* = 127 cells). **i** PMA, expressed as AUC, after periorbital injection of CGRP (1.5 nmol), capsaicin (CPS, 50 pmol) or vehicles in C57BL/6 J male mice pre-treated (0.5 h) with DIPMA-MK-3207, MK-3207 (0.1, 0.3, 1 pmol), DIPMA-Ø or vehicle (*n* = 8 mice per group). Mean±SEM. ***P < 0.001 vs. DIPMA-Ø/Veh, ^###^*P* < 0.001 vs. MK-3207 0.3 pmol and MK-3207 1 pmol. **f**, **g**, **i** 1-way ANOVA, Bonferroni correction. Source data are provided as a Source Data file.

To target CLR in endosomes, we generated self-assembling soft polymer nanoparticles containing a CLR antagonist. Diblock copolymers were synthesized with a hydrophilic shell of P(PEGMA-co-DMAEMA) and a hydrophobic core of P(DIPMA-co-DEGMA) (Fig. 8a). Gel permeation chromatography and ^1^H-nuclear magnetic resonance (^1^H-NMR) confirmed the molecular weight and composition of nanoparticles (Supplementary Fig. 7a–d). Nanoparticles were self-assembled with MK-3207, a potent hydrophobic antagonist of human CLR/RAMP1, forming DIPMA-MK-3207 (Fig. 8a). Empty nanoparticles (DIPMA-Ø) were used as a control. Nanoparticles were uniformly spherical, with the similar diameter (30–35 nm) and ζ-potential (−0.4–1.3 mV) (Fig. 8b, c). DIPMA nanoparticles demonstrate a pH-dependent cargo release at pH < ~6.5, consistent with the protonation of the DIPMA tertiary amine (pK_a_ 6.1), charge repulsion, and disassembly^34^. DIPMA nanoparticles enter cells by clathrin- and dynamin-mediated endocytosis and disassemble in acidic early endosomes^34^. To determine whether DIPMA nanoparticles target endosomes containing CLR/RAMP1, HSCs expressing early endosomal antigen-1-GFP (EEA1-GFP) were incubated with DIPMA-Cy5 for 30 min to allow the accumulation in EEA1-GFP + ve endosomes (Fig. 7d, Supplementary Movie 6). Cells were then incubated with TAMRA-CGRP, which was detected in endosomes containing Cy5-DIPMA within 5–10 min (Fig. 8d, Supplementary Movie 6). Thus, DIPMA nanoparticles accumulate with CLR/RAMP1 in early endosomes of Schwann cells.

To determine whether DIPMA-MK-3207 can antagonize CLR in endosomes, we measured CGRP-stimulated cAMP formation using the CAMYEL cAMP BRET sensor, which detects total cellular cAMP. HEK293T cells expressing rat CLR/RAMP1 (HEK-rCRL/RAMP1) were preincubated with graded concentrations of DIPMA-MK-3207 or free MK-3207, DIPMA-Ø or vehicle (control) for 30 min. Beginning at 0 min, baseline BRET was measured for 5 min, and cells were then challenged with CGRP. At 10 min, cells were washed to remove extracellular CGRP, and BRET was measured up to 35 min. In vehicle-treated cells, CGRP stimulated a prompt increase in cAMP formation (1^st^ phase, 6–10 min) that gradually declined after agonist removal from the extracellular fluid (2^nd^ phase, 11–35 min) (Fig. 8e). DIPMA-Ø did not affect this response. Free MK-3207 and DIPMA-MK-3207 (100, 316 nM) both inhibited CGRP-evoked cAMP in the 1^st^ phase to a similar extent (Fig. 8f). During the 2^nd^ phase, free MK-3207 was inactive at all concentrations whereas DIPMA-MK3207 (31.6, 100, 316 nM) strongly inhibited responses (Fig. 8g). The results suggest that DIPMA-MK3207 can antagonize the sustained phase of CGRP-stimulated formation of cAMP, which is attributable to endosomal CLR/RAMP1 signaling.

To assess the antagonism of the pain signaling pathway in HSCs, we measured CGRP-evoked changes in Ca^2+^ response, which depend on endosomal CGRP signaling and activation of TRPA1. HSCs were preincubated with graded concentrations of DIPMA-MK-3207 or MK-3207 for 20 min to allow the accumulation in endosomes, and washed to remove extracellular compounds. At 10 min after washing, cells were challenged with CGRP and Ca^2+^ response was measured as an index of TRPA1 activity. DIPMA-MK-3207 inhibited CGRP-evoked increase in Ca^2+^ response (IC_50_ 15.4 nM, 95% confidence interval, 10.9–21.0 nM) more potently than free MK-3207 (IC_50_ 2.9 µM, 95% confidence interval, 1.9–4.2 µM, *P* < 0.0001) (Fig. 8h). To assess antinociception, DIPMA-MK-3207 or free MK-3207 (0.1, 0.3, 1.0 pmol) was injected into the periorbital region 30 min before periorbital injection of CGRP or capsaicin. DIPMA-MK-3207 (0.3, 1.0 pmol) more effectively inhibited PMA than the same doses of free MK-3207 (Fig. 8i). DIPMA-Ø had no effect. Thus, endosomal targeting enhances the efficacy of a CLR/RAMP1 antagonist in a preclinical model of migraine pain.

## Discussion

The major findings of the present study are that CGRP causes PMA by activating CLR/RAMP1 of Schwann cells, CLR/RAMP1 signals from endosomes of Schwann cells to activate pain pathways, and endosomal CLR/RAMP1 can be targeted using nanoparticles and endocytosis inhibitors to relieve CGRP-evoked PMA. CLR/RAMP1 stimulation and trafficking to endosomes results in a persistent cAMP-dependent NOS activation and generation of NO, a mediator of migraine pain^19^. The role of NO in PMA is crucial, yet transient, as it is temporally limited to the engagement of TRPA1/NOX1, which releases ROS with a dual function. On one hand, ROS target TRPA1/NOX1 of Schwann cells to maintain ROS generation by a feed-forward mechanism. On the other hand, as suggested by experiments with selective TRPA1 deletion in primary sensory neurons, ROS target TRPA1 on nociceptors to signal allodynia to the CNS.

Periorbital capsaicin injection elicited acute nociception mediated by TRPV1 excitation and ensuing afferent discharge, which signals pain to the CNS. In a larger cutaneous area, capsaicin evoked delayed and prolonged PMA. While the acute pain response is most likely dependent on ion influx associated with TRPV1 activation, the mechanism underlying mechanical hypersensitivity^7,44^ has remained elusive. Our findings support the existence of a paracrine mechanism that underlies PMA associated with neurogenic inflammation. We suggest that capsaicin locally activates TRPV1 + ve nerve fibers to generate action potentials that propagate antidromically into collateral fibers which release CGRP in a broader area, thus eliciting widespread PMA. PMA depends on the interaction between peptidergic nerve fibers, surrounding Schwann cells and nociceptive neurons that convey allodynic signals to the CNS. CGRP liberated from the varicosities of trigeminal TRPV1 + ve nerve fibers binds to CLR/RAMP1 of adjacent Schwann cells.

CNS perturbations may target the trigeminovascular system and initiate the migraine attack^22,23^. These central mechanisms may underlie the delayed facial allodynia associated with migraine^20,21^. However, the beneficial effect of anti-CGRP medicines that do not cross the blood-brain barrier suggests that CGRP acts in the periphery to elicit pain. The peripheral site of the algesic action of CGRP released from peptidergic C-fibers has been proposed as the CLR/RAMP1 on adjacent non-peptidergic Aδ-fibers^45^ and more precisely at the level of the node of Ranvier^28^. The present results in *Adv-Cre*^*+*^*;Ramp1*^*fl/fl*^ mice suggest that CGRP does not act on trigeminal nociceptors to cause PMA in mice. This is consistent with failure of CGRP administration to elicit any itch, pain or axon reflex responses in humans^46^. Instead, our results support the hypothesis that CGRP released from trigeminal nociceptors targets CLR/PAMP1 on Schwann cells that wrap their terminals to evoke PMA. A limitation of our study is that we only assessed PMA that mimics one component of migraine pain. We cannot exclude the possibility that central mechanisms contribute to other pain symptoms of migraine, and that some of the locally administered antagonists used in the present study penetrated the CNS, where they could also influence pain transmission. Although human Schwann cells express CLR/RAMP1 and show functional responses to CGRP that can account for allodynia in mice, further work is needed to understand whether similar mechanisms account for migraine pain in humans.

Another limitation of the present study is that we cannot pinpoint which type of neuron conveys the signals that underlie mechanical allodynia in the trigeminal region. Specifically, we were unable to distinguish between TRPV1-expressing nerve fibers that release CGRP and TRPA1-expressing nerve fibers that are targeted by Schwann cell ROS and convey allodynic signals centrally, since TRPV1 and TRPA1 may coexist in the same population of CGRP-expressing Aδ- or C-fiber primary sensory neurons^2,41^. Most Schwann cells in Remak bundles contain multiple unmyelinated axons from C-fiber nociceptors, including CGRP + ve fibers, which release the bulk of CGRP^2^, and non-peptidergic isolectin B4 + ve fibers^47^. Thus, CGRP-evoked release of ROS from Schwann cells could induce allodynia by targeting TRPA1 on three neuronal subtypes, including the same Aδ- or C-fiber that releases CGRP, a different C-fiber of the same Remak bundle, or a different adjacent Aδ-fiber. The observation that both C-fiber and Aδ-fiber nociceptors contribute to capsaicin-evoked hypersensitivity in humans^48^ supports the hypothesis that both types of neurons^28,45^ are implicated in CGRP-mediated allodynia, thus highlighting the complex neural transmission of mechanical allodynia associated with neurogenic inflammation.

CLR/RAMP1 signals from endosomes by G-protein-mediated mechanisms that activate a subset of compartmentalized signals, including cytosolic protein kinase C and nuclear extracellular signal-regulated kinase; these kinases regulate excitation of spinal neurons and pain transmission^35^. Our results show that CLR/RAMP1 activates Gα_s_, Gα_q_ and Gα_i_ and recruits βARRs in endosomes of Schwann cells, determined by EbBRET. Inhibitors of clathrin- and dynamin-mediated endocytosis blocked the recruitment of CLR/RAMP1, Gα and βARR to endosomes, which presumably requires CLR/RAMP1 endocytosis. GPCR/Gα signaling complexes have also been detected in endosomes by using conformationally selective nanobodies^49^. The observation that endocytosis inhibitors attenuated CGRP-stimulated cAMP formation and activation of NOS and TRPA1 reveals a central role for CLR/RAMP1 signaling in endosomes of Schwann cells in CGRP-evoked periorbital pain. Endocytosis of other Gs-coupled GPCRs is also necessary for the full repertoire of cAMP-mediated signaling outcomes, which entails endosomal recruitment of adenylyl cyclase 9^50^ and assembly of metastable accumulations of PKA^51^. We found that a nanoparticle-encapsulated CLR/RAMP1 antagonist, which targeted CLR/RAMP1 in endosomes and released cargo in the acidified endosomal microenvironment^34^, also attenuated CGRP-stimulated cAMP formation and blunted TRPA1 activation.

The observation that periorbital injection of inhibitors of clathrin and dynamin and of DIPMA-MK-3207 prevented CGRP- and capsaicin-evoked PMA provides the evidence for a prominent role of endosomal CGRP signaling of pain from a peripheral site. The finding that nanoparticle encapsulation enhanced the potency of a CGRP antagonist for inhibition of endosomal signaling and resultant nociception supports the hypothesis that CLR/RAMP1 in endosomes mediates facial allodynia which contributes to migraine pain. Nanoparticle encapsulation similarly boosts the efficacy of an NK_1_ receptor antagonist in preclinical models of inflammatory and neuropathic pain^34^. An antagonist of CLR/RAMP1 conjugated to a membrane lipid cholestanol also accumulates in endosomes and provides superior relief from pain^35^, which reinforces the importance of CLR/RAMP1 endosomal signaling for pain transmission.

Limitations of the present study include uncertainty about the nature of the CLR/RAMP1 signaling complex in endosomes of Schwann cells, which warrants further investigation by proteomics approaches. Although some of the pharmacological inhibitors used to dissect the signaling pathway can have non-specific actions, we bolstered confidence in selectivity by using inhibitors of the same pathway and by genetic deletion of GPCRs and TRP channels. Our findings reveal a prominent role for CLR/RAMP1 in Schwann cells for CGRP-evoked periorbital pain. Future studies will investigate the role of this pathway in preclinical models of migraine pain.

Monoclonal antibodies to CGRP, although beneficial, are not effective in all patients^10^. While non-CGRP-dependent mechanisms might explain this failure^52^, monoclonal antibodies likely do not inhibit CGRP signaling in endosomes. The small molecule CLR/RAMP1 antagonist, rimegepant, was found to resolve migraine attacks in patients treated with the anti-CLR/RAMP1 monoclonal antibody, erenumab^53^. This unexpected result was interpreted by the inherent membrane permeability of the lipophilic antagonist rimegepant^54^ that might favor inhibition of CGRP signaling in endosomes^53^, while neither receptor-targeted nor ligand-targeted monoclonal antibodies internalized with CLR/RAMP1 activated by CGRP^55^. Our results showing a superior inhibition of CGRP signaling in Schwann cells and of PMA by DIPMA-MK-3207, which selectively targets receptor activity in endosomes, reveal a better approach to control allodynia.

**Fig. 9:**
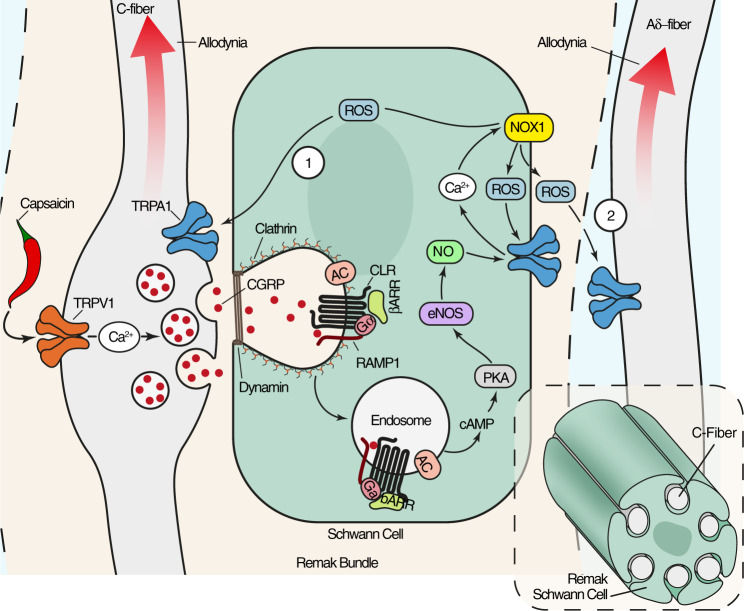
Schematic representation of the pathway that signal prolonged cutaneous allodynia elicited by CGRP released and associated with neurogenic inflammation. The pro-migraine neuropeptide, CGRP, released from trigeminal cutaneous afferents, activates CLR/RAMP1 on Schwann cells. CLR/RAMP1 traffics to endosomes, where sustained G protein signaling increases cAMP and stimulates PKA that results in nitric oxide synthase activation. The ensuing release of nitric oxide targets the oxidant-sensitive channel, TRPA1, in Schwann cells, which elicits persistent ROS generation. ROS triggers TRPA1 on adjacent C- (1) or Aδ-fiber (2) afferents resulting in periorbital allodynia, a hallmark of migraine pain. The inset shows several unmyelinated axons invaginated into a Schwann cell forming a Remak bundle.

In 1936, Sir Thomas Lewis postulated^1^ that in human skin action potentials are carried antidromically from the injured nerve terminal to collateral branches from where a chemical substance is released that produces the flare and increases the sensitivity of other fibers responsible for pain. CGRP has been previously identified as the mediator of neurogenic vasodilatation in rodents^2^, and in humans^8^. Herein, we propose that CGRP is the ‘chemical substance’ that, via the essential role of endosomal CLR/RAMP1, TRPA1/NOX1 and oxidative stress of surrounding Schwann cells, sustains the enhanced sensitivity of primary sensory neurons associated with neurogenic inflammation (Fig. 9). The present results suggest that peripherally acting anti-CGRP medicines reduce migraine pain in part by targeting the facial allodynia that originates from CGRP-mediated endosomal signaling in Schwann cells.

## Methods

The research conducted complies with all relevant ethical regulations. Animal experiments were carried out in accordance with European Union (EU) guidelines for animal care procedures and the Italian legislation (DLgs 26/2014) application of the EU Directive 2010/63/EU. The study was approved by the National Committee for the Protection of Animals used for Scientific Purposes of the Italian Ministry of Health (research permits #383/2019-PR and #765/2019-PR). The use of formalin-fixed paraffin-embedded (FFPE) sections of human abdominal cutaneous tissues was approved by the Local Ethics Committee of the Florence University Hospital (Area Vasta Toscana Centro) (18271_bio/2020), according to the Helsinki Declaration, and all patients gave their informed consent. Participants did not receive any form of compensation. Biopsies of human abdominal skin analyzed derived from three different patients [female, median age 58 years (range 56–61 years)].

### Experimental model and subject details

#### Animals

Male and female mice were used throughout (25–30 g, 5–8 weeks). The following strains of mice were used C57BL/6 J mice (Charles River, RRID:IMSR_JAX:000664); wild-type (*Trpa1*^*+/+*^) and TRPA1-deficient (*Trpa1*^−/−^; B6129P-Trpa1^tm1Kykw^/J; RRID:IMSR_JAX:006401, Jackson Laboratory) mice;^56^ wild-type (*Trpv1*^*+/+*^) and TRPV1-deficient (*Trpv1*^−/−^; B6129X1-Trpv1^tm1Jul^/J, RRID:IMSR_JAX:003770, Jackson Laboratory) mice. Genetically modified mice were maintained as heterozygotes on a C57BL/6 J background.

To generate mice in which the *Trpa1* and *Ramp1* genes were conditionally silenced in Schwann cells/oligodendrocytes, homozygous 129S-*Trpa1*^tm2Kykw^/J (*floxed Trpa1, Trpa1*^*fl/fl*^, RRID:IMSR_JAX:008649 Jackson Laboratory) and C57BL/6N-*Ramp1*^<tm1c(EUCOMM)Wtsi>/H^ (*floxed Ramp1*, *Ramp1*^*fl/fl*^ Stock No: EM:07401, MRC HARWELL Mary Lion Center)^57^ were crossed with hemizygous B6.Cg-Tg(*Plp1-Cre*^*ERT*^)3Pop/J mice (*Plp1-Cre*^*ERT*^, RRID:IMSR_JAX:005975 Jackson Laboratory), expressing a tamoxifen-inducible Cre in myelinating cells (Plp1, proteolipid protein myelin 1)^30^. The progeny (*Plp1-Cre*^*ERT*^;*Trpa1*^*fl/fl*^ and *Ramp1-Cre*^*ERT*^;*Trpa1*^*fl/fl*^) was genotyped by PCR for *Trpa1, Ramp1* and *Plp1-Cre*^*ERT*^. Mice negative for *Plp1-Cre*^*ERT*^ (*Plp1-Cre*^*ERT-*^;*Trpa1*^*fl/fl*^ and *Plp1-Cre*^*ERT-*;^*Ramp1*^*fl/fl*^) were used as control. Both positive and negative mice to *Cre*^*ERT*^ and homozygous for floxed *Trpa1* (*Plp1-Cre*^*ERT*^*;Trpa1*^*fl/fl*^ and *Plp1-Cre*^*ERT-*^*;Trpa1*^*fl/fl*^, respectively) and floxed *Ramp1 (Plp1-Cre*^*ERT*^*;Ramp1*^*fl/fl*^ and *Plp1-Cre*^*ERT-*^*;Ramp1*^*fl/fl*^) mice were treated with 4-hydroxytamoxifen (4-OHT) by subcutaneous periorbital (p.orb.) injection (0.02 mg/10 μl in corn oil once a day for 3 consecutive days). Some *Plp1-Cre*^*ERT*^*;Ramp1*^*fl/fl*^ and *Plp1-Cre*^*ERT-*^*;Ramp1*^*fl/fl*^ mice were treated with intraperitoneal (i.p.) or intraplantar (i.pl.) 4-OHT (1 mg/100 μl or 0.02 mg/10 μl in corn oil once a day for 3 consecutive days, respectively).

Treatments resulted in Cre-mediated ablation of *Trpa1* and *Ramp1* in PLP-expressing Schwann cells/oligodendrocytes. To selectively delete the *Trpa1* and *Ramp1* gene in primary sensory neurons, *Trpa1*^*fl*^*/*^*fl*^ and *Ramp1*^*fl/fl*^ mice were crossed with hemizygous *Advillin-Cre* mice *(Adv-Cre)*^30,58,59^. Both positive and negative mice to *Cre*^*ERT*^ and homozygous for floxed *Trpa1* (*Adv-Cre*^*+*^*;Trpa1*^*fl/fl*^ and *Adv-Cre*^*−*^*;Trpa1*^*fl/fl*^, respectively) and floxed *Ramp1 (Adv-Cre*^*+*^*;Ramp1*^*fl/fl*^ and *Adv-Cre*^*−*^*;Ramp1*^*fl/fl*^) were used.

The group size of *n* = 8 animals for behavioral experiments was determined by sample size estimation using G*Power (v3.1)^60^ to detect size effect in a post-hoc test with type 1 and 2 error rates of 5 and 20%, respectively. Mice were allocated to vehicle or treatment groups using a randomization procedure (http://www.randomizer.org/). Four independent and blinded investigators performed the treatments, behavioral experiments, genotyping and data analysis, respectively. No animals were excluded from experiments.

The behavioral studies followed the animal research reporting in vivo experiment (ARRIVE) guidelines^61^. Mice were housed in a temperature (20 ± 2 °C) and humidity (50 ± 10%) controlled vivarium (12 h dark/light cycle, free access to food and water, five animals per cage). At least 1 h before behavioral experiments, mice were acclimatized to the experimental room and behavior was evaluated between 9:00 am and 5:00 pm. All the procedures were conducted following the current guidelines for laboratory animal care and the ethical guidelines for investigations of experimental pain in conscious animals set by the International Association for the Study of Pain^62^. Animals were anesthetized with a mixture of ketamine and xylazine (90 mg/kg and 3 mg/kg, respectively, i.p.) and euthanized with inhaled CO_2_ plus 10–50% O_2_.

#### Cell lines

Primary cultures of human Schwann cells (HSCs, #1700, ScienCell Research Laboratories) were grown and maintained in Schwann cell medium (#1701, ScienCell Research Laboratories) at 37 °C in 5% CO_2_ and 95% O_2_. Cells were passaged at 90% confluency and discarded after 12 passages. HEK293T (#CRL-3216™, American Type Culture Collection) cells were cultured in Dulbecco’s Modified Eagle’s Medium (DMEM) supplemented with heat-inactivated fetal bovine serum (FBS, 10%), L-glutamine (2 mM), penicillin (100 U/ml) and streptomycin (100 mg/ml) at 37 °C in 5% CO_2_ and 95% O_2_. The mouse Schwann cell line (IMS32 cells, #PMC-SWN-IMS32-COS, Cosmo Bio USA) was grown and maintained in Schwann cell medium (#PMC-SWN-MM-COS, Cosmo Bio USA) at 37 °C in 5% CO_2_ and 95% O_2_^63,64^. All cells were used when received without further authentication.

#### Pharmacological reagents

Supplementary Table 1 and 2 provide doses, routes of administration and concentrations and of all pharmacological reagents.

### Behavioral experiments

#### Treatment protocol

Subcutaneous injections were made in the periorbital area 2–3 mm from the external eyelid corner^17^. Briefly, the mouse was lifted by the base of the tail and placed on a solid surface with one hand and the tail was pulled back. Then, it was quickly and firmly picked up by the scruff of the neck with the thumb and index finger of the other hand. The injection was made rapidly by a single operator with minimal animal restraint. Mice received unilateral (right side) injections (10 μl/site) of CGRP (1.5 nmol in 0.9% NaCl), SP (3.5 nmol in 0.9% NaCl), capsaicin (10, 50, 100 pmol in 0.1% dimethyl sulfoxide, DMSO), or vehicles (control). Mice received bilateral injections (10 µl/site, right side same site as stimulus, left side symmetrical to right side) of antagonists and inhibitors. CGRP (1.5 nmol in 0.9% NaCl) or vehicle was also administered by intraplantar (i.pl., 20 μl/site) or systemic (0.1 mg/kg, i.p.) injection. GTN was administered at 10 mg/kg, i.p. injection.

#### Acute nociception

Immediately after the p.orb. injection, mice were placed inside a plexiglass chamber and spontaneous nociception was assessed for 10 min by measuring the time (s) that the animal spent rubbing the injected area of the face with its paws^17,65^.

#### Periorbital mechanical allodynia

PMA was assessed using the up-down paradigm^66,67^ in the same mice in which acute nociceptive responses were monitored. Briefly, mice were placed in a restraint apparatus designed for the evaluation of periorbital mechanical thresholds^17^. One day before the first behavioral observation, mice were habituated to the apparatus. PMA was evaluated in the periorbital region over the rostral portion of the eye (i.e., the area of the periorbital region facing the sphenoidal rostrum)^68^ before (basal threshold) and after (0.5, 1, 2, 4, 6, 8 h) treatments. On the day of the experiment, after 20 min of adaptation inside the chamber, a series of 7 von Frey filaments in logarithmic increments of force (0.02, 0.04, 0.07, 0.16, 0.4, 0.6, and 1.0 g) were applied to the periorbital area perpendicular to the skin, with sufficient force to cause slight buckling, and held for approximately 5 s to elicit a positive response. Mechanical stimuli were applied homolaterally outside the periorbital area at a distance of 6–8 mm from the site where stimuli were injected. The response was considered positive by the following criteria: mouse vigorously stroked its face with the forepaw, head withdrawal from the stimulus, or head shaking. Mechanical stimulation started with the 0.16 g filament. The absence of response after 5 s led to the use of a filament with increased force, whereas a positive response led to the use of a weaker (*i.e*. lighter) filament. Six measurements were collected for each mouse or until four consecutive positive or negative responses occurred. The 50% mechanical withdrawal threshold (expressed in g) was then calculated from these scores by using a δ value of 0.205, previously determined.

#### Paw mechanical allodynia

Paw mechanical allodynia was evaluated by measuring the paw withdrawal threshold by using the up-down paradigm^66,67^. Mice were acclimatized (1 h) in individual clear plexiglass boxes on an elevated wire mesh platform, to allow for access to the plantar surfaces of the hind paws. von Frey filaments of increasing stiffness (0.07, 0.16, 0.4, 0.6, and 1.0, 1.4 and 2 g) were applied to the hind paw plantar surfaces of mice with enough pressure to bend the filament. The absence of a paw being lifted after 5 s led to the use of the next filament with an increased force, whereas a lifted paw indicated a positive response, leading to the use of a subsequently weaker filament. Six measurements were collected for each mouse or until four consecutive positive or negative responses occurred. The 50% mechanical withdrawal threshold (expressed in g) was then calculated.

#### Primary culture of mouse Schwann cells

Mouse Schwann cells (MSC) were isolated from sciatic or trigeminal nerves of C57BL/6 J, and from sciatic nerve of *Trpa1*^*+/+*^ and *Trpa1*^−/−^, *Plp1-Cre*^*ERT+*^*;Ramp1*^*fl/fl*^ and *Plp1-Cre*^*ERT-*^*;Ramp1*^*fl/fl*^ mice^30,69^. The epineurium was removed, and nerve explants were divided into 1 mm segments and dissociated enzymatically using collagenase (0.05%) and hyaluronidase (0.1%) in Hank’s Balanced Salt Solution (HBSS, 2 hr, 37 °C). Cells were collected by centrifugation (150xg, 10 min, room temperature) and the pellet was resuspended and cultured in DMEM containing fetal calf serum (10%), L-glutamine (2 mM), penicillin (100 U/ml), streptomycin (100 mg/ml), neuregulin (10 nM) and forskolin (2 μM). Three days later, cytosine arabinoside (Ara-C, 10 mM) was added to remove fibroblasts. Cells were cultured at 37 °C in 5% CO_2_ and 95% O_2_. The culture medium was replaced every 3 days and cells were used after 15 days of culture.

#### qRT-PCR

Total RNA was extracted from HSCs, IMS32 and sciatic or trigeminal MSCs cells using the RNeasy Mini kit (Qiagen SpA), according to the manufacturer’s protocol. RNA concentration and purity were assessed spectrophotometrically by measuring the absorbance at 260 nm and 280 nm. RNA was reverse transcribed with the Qiagen QuantiTect Reverse Transcription Kit (Qiagen SpA) following the manufacturer’s protocol. For mRNA relative quantification, rt-PCR was performed on Rotor Gene® Q (Qiagen SpA, Rotor-Gene® Q- Software Version 2.3.1.49). The relative abundance of mRNA transcripts was calculated using the delta CT method and normalized to GAPDH levels. The sets of primers for human and mouse cells are listed in the Supplementary Table 3.

#### Calcium imaging

HSCs, IMS32 and sciatic nerve MSCs cells were plated on poly-L-lysine-coated (8.3 μM) 35 mm glass coverslips and maintained at 37 °C in 5% CO_2_ and 95% O_2_ for 24 h. Cells were loaded (40 min) with Fura-2 AM-ester (5 μM) added to the buffer solution (37 °C) containing (in mM) 2 CaCl_2_; 5.4 KCl; 0.4 MgSO_4_; 135 NaCl; 10 D-glucose; 10 HEPES and bovine serum albumin (BSA, 0.1%) at pH 7.4. Cells were washed and transferred to a chamber on the stage of a fluorescent microscope for recording (Olympus IX 81) and exposed to CGRP (0.01–10 μM) or vehicle (0.9% NaCl) and the Ca^2+^ response was monitored for approximately 40 min. In another set of experiments, IMS32 cells were exposed to AITC (30 μM) or vehicle (0.03% DMSO). The Ca^2+^ response to CGRP was monitored in the presence of CGRP8-37 (100 nM), olcegepant (100 nM), SQ22536 (100 μM), L-NAME (10 μM), A967079 (50 μM), PBN (50 μM), H89 (1 μM), ML171 (1 μM) or vehicle (0.1 % DMSO), and in the presence of DIPMA-MK-3207 (1–1000 nM) and MK-3207 free drug (0.01–1000 μM) or DIPMA-empty. Cells were preincubated with DIPMA-MK-3207 or MK-3207 for 20 min at room temperature to allow nanoparticle accumulation in endosomes. Some experiments used Ca^2+^-free buffer solution containing EDTA (1 mM). Results were expressed as percent increase in ratio_340/380_ over baseline normalized to the maximum effect induced by ionomycin (5 μM) added at the end of each experiment.

#### Protein extraction and western immunoblot assay

HSCs and IMS32 cells were plated on 60 mm culture dish and maintained in 5% CO_2_ and 95% O_2_ (37 °C, until confluence). HSCs and IMS32 cells were homogenized in RIPA buffer [NaCl (150 mM), Tris-base (50 mM), EGTA (5 mM), Triton X-100 (1%), sodium deoxycholate (0.5%), sodium dodecyl sulfate (0.1%)] containing dithiothreitol (1 mM) and complete protease inhibitor cocktail. Lysates were centrifuged at 16,128x *g* at 4 °C for 10 min. Protein concentration in supernatants was determined using BCA protein assay (Thermo Scientific). Samples with equal amounts of proteins (20 μg) were then separated by NuPAGE 4–12% bis-tris gel electrophoresis (Life Technologies), and the proteins were transferred to a nitrocellulose (Bio-Rad). Membranes were incubated with dry milk (5%) in Tris buffer [(TBST; Tris (20 mM) at pH 7.5, NaCl (150 mM)] containing Tween 20 (0.1%) for 1 h at room temperature, and incubated with the following primary antibodies: RAMP1 [ERP10867] (#ab156575, rabbit monoclonal, 1:1000, Abcam, Lot: GR3196403-5), CRLR (#NBP1-59073, rabbit polyclonal, 1:500, Novus Biological, Lot: 8312) or β-actin (#ab6276, mouse monoclonal, 1:5000, Abcam, Lot: GR 141-7) at 4 °C overnight. Membranes were then probed with goat anti-mouse or donkey antirabbit IgG conjugated with horseradish peroxidase (HRPO, 1:10,000, Bethyl Laboratories Inc., Cat#A90-516P and Cat#A120-208P) for 2 hr at room temperature. Finally, membranes were washed three times with TBST, and bound antibodies were detected using chemiluminescence reagents (Pierce™ ECL, Thermo Scientific), and revealed using an imaging system (ChemiDoc version 2.3.0.07 BioRad). The density of specific bands was measured using an image processing program (ImageJ 1.32 J, National Institutes of Health) and normalized to β-actin.

#### In-cell ELISA assay

HSCs or IMS32 cells were plated in 96-well black wall clear bottom plates (Corning Life Sciences) (5 × 10^5^ cells/well) and maintained at 37 °C in 5% CO_2_ and 95% O_2_ for 24 h. HSCs and IMS32 cells were exposed to CGRP (1 and 10 μM, respectively) or its vehicle (phosphate-buffered saline, PBS) for 5, 10, 15, 30 and 60 min, at 37 °C, then washed with DMEM pH 2.5 and fixed in 4% paraformaldehyde for 30 min. Cells were then washed with TBST (0.05%) and blocked with donkey serum (5%) for 4 h at room temperature and incubated overnight 4 °C with eNOS^pS1177^ (#ab184154, rabbit polyclonal, 1:100, Abcam, Lot: GR3257047-9). Cells were then washed and incubated with donkey anti-rabbit IgG conjugated with horseradish peroxidase (HRPO, 1:2000, Bethyl Laboratories Inc.) for 2 h at room temperature. Cells were then washed and stained using SIGMAFAST OPD for 30 min protected from light. After the incubation period, the absorbance was measured at 450 nm. Change in NOS3 phosphorylation was calculated as a percentage of the signal in vehicle-treated cells.

#### cAMP ELISA assay

cAMP level was determined by the CatchPoint™ cyclic-AMP fluorescent assay kit (#R8088, Molecular Device) according to the manufacturer’s protocol. Briefly, HSCs or IMS32 cells were plated in 96-well black wall clear bottom plates (Corning Life Sciences) (5 × 10^5^ cells/well) and maintained in 5% CO_2_ and 95% O_2_ (24 h, 37 °C). The cultured medium was replaced with HBSS added with olcegepant (100 nM), CGRP8-37 (100 nM), SQ22536 (100 μM), L-NAME (10 μM) or vehicle (0.1% DMSO in HBSS) for 20 min at room temperature. HSCs or IMS32 cells were then stimulated with CGRP (1 and 10 μM, respectively), forskolin (1 μM, positive control) or their vehicles (HBSS) and maintained for 40 min at room temperature protected from light. Signal was detected 60 min after exposure to the stimuli. cAMP level was calculated using cAMP standards and expressed as nmol/1.

#### Nitric oxide assay

Nitric oxide was determined by using the fluorometric-orange assay kit (#ab219932, Abcam) according to the manufacturer’s protocol. HSCs or IMS32 cells were plated 96-well black wall clear bottom plates (Corning Life Sciences) (5 × 10^5^ cells/well) and maintained in 5% CO_2_ and 95% O_2_ (24 h, 37 °C). The cultured medium was replaced with Hanks’ balanced salt solution (HBSS) added with olcegepant (100 nM), CGRP8-37 (100 nM), SQ22536 (100 μM), L-NAME (10 μM), A967079 (50 μM), PS2 or Dy4 (both 30 µM), PS2 inact or Dy4 inact (30 µM) or vehicle (0.1% DMSO in HBSS) for 20 min at room temperature. HSCs or IMS32 cells were then stimulated with CGRP (1 and 10 μM, respectively), diethylamine NONOate (1 mM, positive control) or their vehicles (HBSS) and maintained for 40 min at room temperature protected from light. Signal was detected 60 min after exposure to the stimuli. Change in nitric oxide level was calculated as percentage of the vehicle.

#### H_2_O_2_ assay

H_2_O_2_ was determined by using the Amplex Red assay (#A12222, Thermo Fisher, Invitrogen). HSCs or IMS32 cells were plated in 96-well black wall clear bottom plates (Corning Life Sciences) (5 × 10^5^ cells/well) and maintained in 5% CO_2_ and 95% O_2_ (24 h, 37 °C). The cultured medium was replaced with Krebs-Ringer phosphate (KRP, composition in mM: 2 CaCl_2_; 5.4 KCl; 0.4 MgSO_4_; 135 NaCl; 10 D-glucose; 10 HEPES [pH 7.4]) added with olcegepant (100 nM), CGRP8-37 (100 nM), SQ22536 (100 μM), L-NAME (10 μM), A967079 (50 μM), PBN (50 μM) or vehicle (0.1% DMSO in KRP) for 20 min at room temperature. HSCs or IMS32 cells were then stimulated with CGRP (1 and 10 μM, respectively) or its vehicle (KRP) added with Amplex red (50 μM) and horseradish peroxidase (HRP, 1 U/ml) and maintained for 40 min at room temperature protected from light. Some experiments were performed in Ca^2+^-free KRP containing EDTA (1 mM). Signal was detected 60 min after exposure to the stimuli. H_2_O_2_ release was calculated using H_2_O_2_ standards and expressed as nmol/1.

#### Immunofluorescence

Anesthetized mice were transcardially perfused with PBS and 4% paraformaldehyde. Trigeminal and sciatic nerves were removed, postfixed for 24 h, and paraffin-embedded. Human and mouse formalin-fixed paraffin-embedded (FFPE) sections (5 μm) were incubated with primary antibodies: TRPA1 (#ab58844, rabbit polyclonal, 1:400, Abcam, Lot: GR165165-21), S100 (#ab14849, mouse monoclonal [4B3], 1:300, Abcam, Lot: GR3233892-2), CLR (#NLS6731, rabbit polyclonal, 1:30, Novus Biologicals, Lot: QC58878-190422), RAMP1- Alexa Fluor 594 (#ab241335, rabbit polyclonal, 1:200, Abcam Lot: GR3247267-4), diluted in fresh blocking solution (PBS, pH 7.4, 2.5% normal goat serum, [NGS]). Sections were then incubated with the fluorescent polyclonal secondary antibodies Alexa Fluor 488 and 555 (#A32731, Lot: VA295501, #A32727, Lot: UL287768, 1:600; Invitrogen), and coverslipped using the mounting medium with DAPI (Abcam). Images were acquired by a Zeiss Axio lmager 2, Zeiss ZEN imaging 2020. The Pearson correlation (Rcoloc) value for RAMP1 and S100 in the colocalization studies were calculated using the colocalization Plugin of the ImageJ software (ImageJ 1.32 J, National Institutes of Health).

#### cAMP cADDIS assay

HSCs were plated on poly-D-lysine-coated 96-well black wall clear bottom plates (Corning Life Sciences) (25 × 10^3^ cells/well) and incubated in 5% CO_2_ and 95% O_2_ for 4–6 h. HSCs were transduced with the baculovirus mediated Green Upward cADDIS cAMP reporter (25 µl/well, Montana Molecular) following manufacturer’s instructions, and cells were incubated in 5% CO_2_ and 95% O_2_ (48 h, 37 °C). HSCs were washed twice in HBSS plus HEPES (10 mM) pH 7.4. Cells were incubated in HBSS/HEPES with the CLR/RAMP1 antagonists olcegepant (100 pM–100 µM) or vehicle (control) for 30 min. Plates were mounted in a FlexStation3 plate reader (Molecular Devices) and fluorescence (485–500 excitation, 515–530 emission with cutoff at 510) was monitored. The baseline was measured for 1 min, and cells were stimulated with human CGRPα (100 pM-10 µM) or forskolin (10 µM, positive control). For single-cell imaging, HSCs were plated on poly-D-lysine-coated 35 mm glass-bottom dishes (MatTek, Ashland) (40 × 10^3^ cells/dish) and incubated in 5% CO_2_ and 95% O_2_ (overnight, 37 °C). HSCs cells were transduced with Green Upward cADDIS and incubated for 48 h. HSCs were washed twice in HBSS/HBS and mounted on a Leica DMI8 microscope (Wetzlar, Germany). Fluorescence (470/40 excitation, 527/30 emission) was measured every 5 s. The baseline was measured for 30 s, and HSCs were challenged with human CGRPα (100 nM). Images were analyzed with ImageJ (NIH). To inhibit endocytosis, cells were incubated in HBSS containing 0.45 M sucrose or normal HBSS (control) for 30 min at 37 °C before cAMP assays.

#### CAMYEL BRET cAMP assay

HEK293T cells stably expressing the CAMYEL BRET sensor (~2 × 10^6^) were seeded into 90 mm Petri dish (Corning™, USA) in DMEM/FBS/Geneticin and incubated in 5% CO_2_ and 95% O_2_ (24 h, 37 °C). Prior to the transfection, the medium was changed to fresh DMEM/FBS/Geneticin and rat CLR/RAMP1 was transfected (2.5 µg CLR/RAMP1 DNA/dish) using JetPEI (Polyplus Transfection, France) at a 1:6 ratio. After 24 h, cells were plated in poly-L-lysine coated black 96 well CulturPlate (Perkin Elmer, USA) and incubated in 5% CO_2_ and 95% O_2_ (24 h, 37 °C). BRET was assessed using a LUMIstar (BMG LABTECH, Germany). On the day of the assay, cells were equilibrated in HBSS for 30 min, supplemented with 12 mM HEPES at 37 °C in CO_2_-free incubator. DIPMA-MK-3207 or free MK-3207 was incubated for 25 min, followed by the addition of coelentrazine-h (50 µM) for 5 min. Baseline was then measured for 5 min, followed by stimulation with CGRP (100 nM, ~EC_50_), vehicle (HBSS) or forskolin (1 µM, positive control), and further measurements for 5 min. Buffer was then replaced by HBSS with coelentrazine-h (50 µM) and measurements were resumed for further 25 min.

#### Synthesis of TAMRA-CGRP and endocytosis of TAMRA-CGRP and Cy5-DIPMA nanoparticles

TAMRA-CGRP was synthesized by JPT Peptide Technologies (Berlin, Germany). A lysine residue conjugated to TAMRA was added to the C-terminus of human CGRPα (A1CDTATCVTHRLAGLLSRSGGVVKNNFVPTNVGSKAF37-K-TAMRA). Peptide purity was > 94%. HSCs were plated on poly-D-lysine-coated 35 mm glass-bottom dishes (MatTek) (50 × 10^3^ cells/dish) and incubated in 5% CO_2_ and 95% O_2_ (overnight, 37 °C). HSCs were transduced with the BacMan CellLight Early Endosomes Rab5a-GFP marker (5 µl/dish) and incubated in 5% CO_2_ and 95% O_2_ (48 h, 37 °C). HSCs were washed twice in HBSS/HEPES. Cells were incubated with the endocytosis inhibitors PS2 or Dy4 (both 30 µM), PS2 inact or Dy4 inact (30 µM), sucrose (0.45 M) or vehicle (control) for 30 min. Cells were imaged using Leica SP8 confocal microscope (Wetzlar), LAS X imaging software. Images were captured every 15 s. Baseline fluorescence was recorded for 2 min. TAMRA-CGRP (1 µM) was added and cells were imaged for 30 min. TAMRA-CGRP internalization to early endosomes at 30 min was quantified using ImageJ (National Institutes of Health). Regions of interest (ROIs) were defined by thresholding the channel for early endosomes (GFP) and particles were analyzed for size (≥0.2 µm^2^) and for roundness (0.2–1). ROIs were then overlayed on the TAMRA-CGRP channel and mean fluorescence was quantified for each ROI. Data are presented as relative fluorescent units (RFUs) per endosome and percentage of CGRP- + ve endosomes (endosomes with TAMRA-CGRP fluorescence >350 RFU) to total endosomes. Imaging experiments were repeated 5–7 times per group and a minimum of 4 cells were imaged for each experiment. To assess the internalization nanoparticles, HSCs were plated as described and incubated with DIPMA-Cy5 nanoparticles (40–60 µg/ml) for 1 h. Cells were washed and imaged as stated.

#### EbBRET assays of G protein and βARR recruitment to the plasma membrane and endosomes cDNAs

Mini-G proteins coupled to Venus were from N. A. Lambert (Augusta University). Constructs were modified to replace Venus with Rluc8. *Renilla* (R) GFP-CAAX (prenylation CAAX box of KRas), tdRGFP-Rab5a, Rluc2-βARR2 and human CLR-Rluc8 were from M. Bouvier (Université de Montréal).

#### Transfection

For EbBRET assays of trafficking of mini-G proteins to the plasma membrane or early endosomes, HEK293T cells were transfected using JetPEI (Polyplus Transfection, France) with bicistronic human myc-CLR-NanoBiT/HA-RAMP1 (0.2 µg) (NanoBiT: GVTGWRLCERILA) and Rluc8-mGα_si/s/sq_ (0.1 µg)^70,71^ and either RGFP-CAAX (0.2 µg)^72^ for cell surface activation or tdRGFP-Rab5a (0.15 µg) for activation in early endosomes. Schwann cells were transfected using lipofectamine 3000 with bicistronic human myc-CLR-NanoBiT/HA-RAMP1 (1 µg) and Rluc8-mGα_si/s/sq_ (2 µg) and either RGFP-CAAX (1.5 µg) or tdRGFP-Rab5a (1.5 µg). For EbBRET assays of recruitment of βARR2^72^ to the plasma membrane or early endosomes, HEK293T cells were transfected with JetPEI with bicistronic human myc-CLR-NanoBiT/HA-RAMP1 (0.2 µg) and Rluc2-βARR2 (0.1 µg) and RGFP-CAAX (0.2 µg) for plasma membrane recruitment or tdRGFP-Rab5a (0.15 µg) for early endosome recruitment. Schwann cells were transfected using lipofectamine 3000 with bicistronic human myc-CLR-NanoBiT/HA-RAMP1 (1 µg) and Rluc2-βARR2 (2 µg) and RGFP-CAAX (1.5 µg) or tdRGFP-Rab5a (1.5 µg). For EbBRET assay of trafficking of CLR from the plasma membrane to early endosomes, HEK293T cells were transfected using JetPEI with human myc-CLR-Rluc8 (0.7 µg), human myc-RAMP1 (0.5 µg) and either plasma membrane marker RGFP-CAAX (0.2 µg) or early endosome marker tdRGFP-Rab5a (0.15 µg).

#### BRET assays

HEK293T cells and HSCs were washed with HBSS buffer complemented with 10 mM HEPES at pH 7.4. Prolume Purple Coelenterazine (2.5 µM, NanoLight Technology) was added, and cells were incubated for 5 min at 37 °C. EbBRET was recorded for 22.5 min in a Synergy Neo2 Microplate reader (BioTek) (acceptor filter: 515 ± 30 nm; donor filter: 410 ± 80 nm). The baseline was measured for 2.5 min, and cells were challenged with human CGRPα (100 nM) or vehicle. ΔBRET represents the EbBRET signal in the presence of agonist subtracted by the EbBRET signal in the presence of the vehicle. To inhibit endocytosis, cells were incubated in HBSS containing 0.45 M sucrose or normal HBSS (control) for 30 min at 37 °C before EbBRET assays.

### Synthesis and Characterization of Nanoparticles

#### Synthesis of P(PEGMA-co-DMAEMA)-b-P(DIPMA-co-DEGMA)

The polymer was synthesized in a two-step sequential polymerization by the reversible addition-fragmentation chain (RAFT) method^73^, as previously described^34^. First, the hydrophilic block, P(PEGMA-co-DMAEMA), was synthesized using the RAFT agent, 2-cyanoprop-2-yl dithiobenzoate (CPBD, 0.0736 g, 3.34 × 10^−4^ mol) and the initiator azobisisobutyronitrile (AIBN, 0.0054 g, 3.34 × 10^−5^ mol) in a ratio of 1:0.1. The monomers poly(ethylene glycol) monomethyl ether methacrylate (PEGMA, 6 g, 0.02 mol) and 2-[*N,N*-(dimethylamino)ethyl] methacrylate (DMAEMA, 0.314 g, 0.001 mol) were used at a ratio of 10:1. The mixture, dissolved in toluene, was deoxygenated by sparging with nitrogen and left to react at 70 °C, 75x *g* for 21 h. The remaining monomers were removed by dialysis (MWCO 3500, Membrane Filtration Products) against acetone for 96 h and the final product was dried for 24 h in a vacuum oven at 37 °C and 1 mbar.

The chain extension reaction to incorporate the monomers 2-[*N,N*-(diisopropylamino)ethyl] methacrylate (DIPMA) and di(ethylene glycol) methyl ether methacrylate (DEGMA) was initiated by AIBN (0.0009 g, 5.35 × 10^−6^ mol), using the hydrophilic block P(PEGMA-co-DMAEMA, 0.4455 g, 3.56 × 10^−5^ mol) and the monomers DIPMA (0.7603 g, 3.56 × 10^−3^ mol) and DEGMA (0.0738 g, 3.92 × 10^−4^ mol), at a ratio of 0.15:1:100:11. The mixture was dissolved in toluene, deoxygenate d and left to react at 70 °C, 75 × *g* for 17.5 h. The final product, P(PEGMA-co-DMAEMA)-b-P(DIPMA-co-DEGMA), was purified as described above.

#### Synthesis of P(PEGMA-co-DMAEMA)-b-P(DIPMA-co-DEGMA-co-Cy5)

The chain extension of P(PEGMA-co-DMAEMA, 0.5 g, 3.85 × 10^−5^ mol) was done in toluene by adding DIPMA (0.82 g, 3.85 × 10^−3^ mol), DEGMA (0.080 g, 4.23 × 10^−4^ mol) and 4,4-dimethyl-2-vinyl-2-oxazolin-5-one (VDM, 0.027 g, 1.92 × 10^−4^ mol), in the presence of AIBN (1 mg, 5.77 × 10^−6^ mol) at a ratio of 1:100:11:5:0.15. The mixture was deoxygenated and left to react at 70 °C, 75 xg for 18 h. Cy5 coupling was performed by mixing 250 μl of the reaction with Cyanine5 amine (Cy5, 0.008 g, 1.20 × 10^−5^ mol). The mixture was left to react at room temperature, 75x *g* for 72 h under dark conditions and the final product was purified as described above.

### Analysis of diblock copolymer

#### Gel permeation chromatography (GPC)

The molecular weights of polymers were determined by GPC using a liquid chromatography system equipped with a (RID-10A) differential refractive index detector (**λ** = 633 nm) and SPD-20A ultraviolet detector (Shimadzu). Samples were fractionated using 5.0 μm bead-size guard column (50 × 7.8 mm) and three Shodex KF-805L columns (300 × 8 mm, 10 μm *n* = bead-size, 5000 Å pore size) in series at 40 °C and eluted in *N,N*-dimethylacetamide (DMAC, HPLC grade, with 0.03% w/v LiBr) at a flow rate of 1 ml/min. A molecular weight calibration curve was produced using polystyrene standards ranging from 500 to 2 × 10^6^ Da.

#### Proton-nuclear magnetic resonance (^1^H-NMR)

The conversion and composition of polymers were assessed by ^1^H-NMR using a Bruker Avance III 400 Ultrashield Plus spectrometer (USA) at 400 mHz running Topspin, version 1.3 and deuterated chloroform (chloroform-d) as solvent. Conversions (Conv%) and repeating monomer units (n) were calculated using peak integrals (*I*) where the subscript number indicates the location of the peak in ppm (I_x_). The Conv% were calculated using the ^1^H-NMR spectra before starting the reactions (*t* = 0) and after the reactions were stopped (*t* = *f*) (^1^H-NMR spectra not shown), with ${Conv} \% =\left(1-\left(\frac{{I}_{6.24(t=f)}}{{I}_{6.24(t=0)}}\right)\right)\times 100$. For P(PEGMA-co-DMAEMA), *n* was calculated using the ^1^H-NMR spectra with ${n}_{{PEGMA}}=\frac{{I}_{4.25}\,-\,{I}_{2.5}}{{I}_{7.8\,-\,7.9}}$ and ${n}_{{DMAEMA}}=\frac{{I}_{2.5}}{{I}_{7.8\,-\,7.9}}$ . For P(PEGMA-co-DMAEMA)-b-(DIPMA-co-DEGMA), n were calculated using the ^1^H-NMR spectra with ${n}_{{DIPMA}}=\frac{{I}_{3}\,+\,{I}_{2.6}}{2\,\times\, {I}_{4-4.}}\times ({n}_{{PEGMA}}+{n}_{{DMAEMA}})$ and ${n}_{{DEGMA}}=\frac{{I}_{3.396}}{{I}_{3.378}}\times {n}_{{PEGMA}}$ (Supplementary Fig. 6) For P(PEGMA-co-DMAEMA)-b-(DIPMA-co-DEGMA-co-VDM), *n* was calculated as described for P(PEGMA-co-DMAEMA)-b-(DIPMA-co-DEGMA) (^1^H-NMR spectra not shown).

#### Self-assembly of nanoparticles (NPs)

The diblock copolymer, P(PEGMA-co-DMAEMA)-b-P(DIPMA-co-DEGMA) was used to self-assemble pH-responsive NPs. For the self-assembly of NPs loaded with MK-3207 (DIPMA- MK-3207) a mixture of 5 mg of the diblock copolymer and 53.5 µg of MK-3207 was dissolved in 0.5 ml of dimethylformamide (DMF). Empty NPs (DIPMA-Ø) were self-assembled without adding MK-3207. The mixture was then added into 4.5 ml of PBS under stirring at a flow rate of 1.2 ml/h, using a syringe pump (Harvard Apparatus). Assemblies of DIPMA-MK-3207 and DIPMA-Ø were dialyzed against PBS for 24 h (MWCO 3500, Membrane Filtration Products). The assembly of NPs for live-cell imaging and biodistribution studies was done as described for DIPMA-Ø using the diblock copolymer P(PEGMA-co-DMAEMA)-b-P(DIPMA-co-DEGMA-co-Cy5), which couples Cy5 on the hydrophobic block, resulting in NPs with Cy5 incorporated in the core (DIPMA-Cy5).

### Characterization of NPs

#### Dynamic light scattering (DLS)

The size distribution of DIPMA-MK-3207 and DIPMA-Ø (1 mg/ml) was determined by DLS (Zetasizer Nano ZS ZEN3600 particle size analyser), using polystyrene cuvettes at 25 °C and 173° backscatter angle.

#### Ultra-performance liquid chromatography-mass spectrometry (LC-MS)

MK-3207 loading into the core of NPs was assessed by LC-MS using a Waters Micromass Quattro Premier triple quadrupole mass spectrometer coupled to a Waters Acquity UPLC (USA). Freeze-dried DIPMA- MK-3207 (1 ml, 1 mg/ml) were dissolved in a mixture of DMSO and formic acid 0.1% (5:2). The samples were prepared for analysis by mixing an aliquot of each preparation with internal standard solution (diazepam, 5 µg/ml) in a 5:2 proportion and made up to 500 µl with the dilution solvent (acetonitrile 50%: formic acid 0.1%, 1:1). Samples were fractionated on a Supelco Ascentis Express RP Amide column (50 mm by 2.1 mm, 2.7 µm particle size) equipped with a Phenomenex SecurityGuard precolumn fitted with a Synergi Polar cartridge, maintained at 40 °C. MK-3207 loading was quantified against MK-3207 standards (0.016–20 µM). Compounds were eluted under gradient conditions with a mobile phase of formic acid (0.05%) and acetonitrile. Mass spectrometry was conducted in positive electrospray ionization conditions and elution of compounds were monitored with multiple-reaction monitoring.

#### Transmission electron microscopy (TEM)

The morphology of NPs was determined by TEM imaging using a Tecnai F20 transmission electron microscope at an accelerating voltage of 120 kV at room temperature. Carbon-coated grids were prepared by plasma discharge (35 s). DIPMA-MK-3207 samples (5 µl, 1 mg/ml) were placed on the grid for 20 s. Samples were negatively stained with uranyl acetate (5 µl, 0.5 wt %, 25 s).

#### Statistical analysis

Results are expressed as mean ± standard error of the mean (SEM). For multiple comparisons, a one-way analysis of variance (ANOVA) followed by the post-hoc Bonferroni’s test or Dunnett’s test was used. Two groups were compared using Student’s *t*-test. For behavioral experiments with repeated measures, the two-way mixed model ANOVA followed by the post-hoc Bonferroni’s test was used. Statistical analyses were performed on raw data using Graph Pad Prism 8 (GraphPad Software Inc.). IC_50_ values and confidence intervals were determined from non-linear regression models using Graph Pad Prism 8 (GraphPad Software Inc.). *P* values less than 0.05 (*P* < 0.05) were considered significant. Statistical tests used and the sample size for each analysis are listed in the Fig. legend.

### Reporting Summary

Further information on research design is available in the Nature Research Reporting Summary linked to this article.

## Supplementary information

Supplementary Information

Description of Additional Supplementary Files

Supplementary Video 1

Supplementary Video 2

Supplementary Video 3

Supplementary Video 4

Supplementary Video 5

Supplementary Video 6

Supplementary Data 1

Reporting Summary

## Data Availability

Source data are provided with this paper.

## Source data

Source data file
