# Supplementary material for: Schwann cell endosome CGRP signals elicit periorbital mechanical allodynia in mice
Source: Nat Commun. 2022 Feb 3;13:646. doi: 10.1038/s41467-022-28204-z (PMC8813987; doi:10.1038/s41467-022-28204-z)
Supplement: Supplementary file 1 — Supplementary Information [file 41467_2022_28204_MOESM1_ESM.pdf]

Supplementary Information

Supplementary Figures

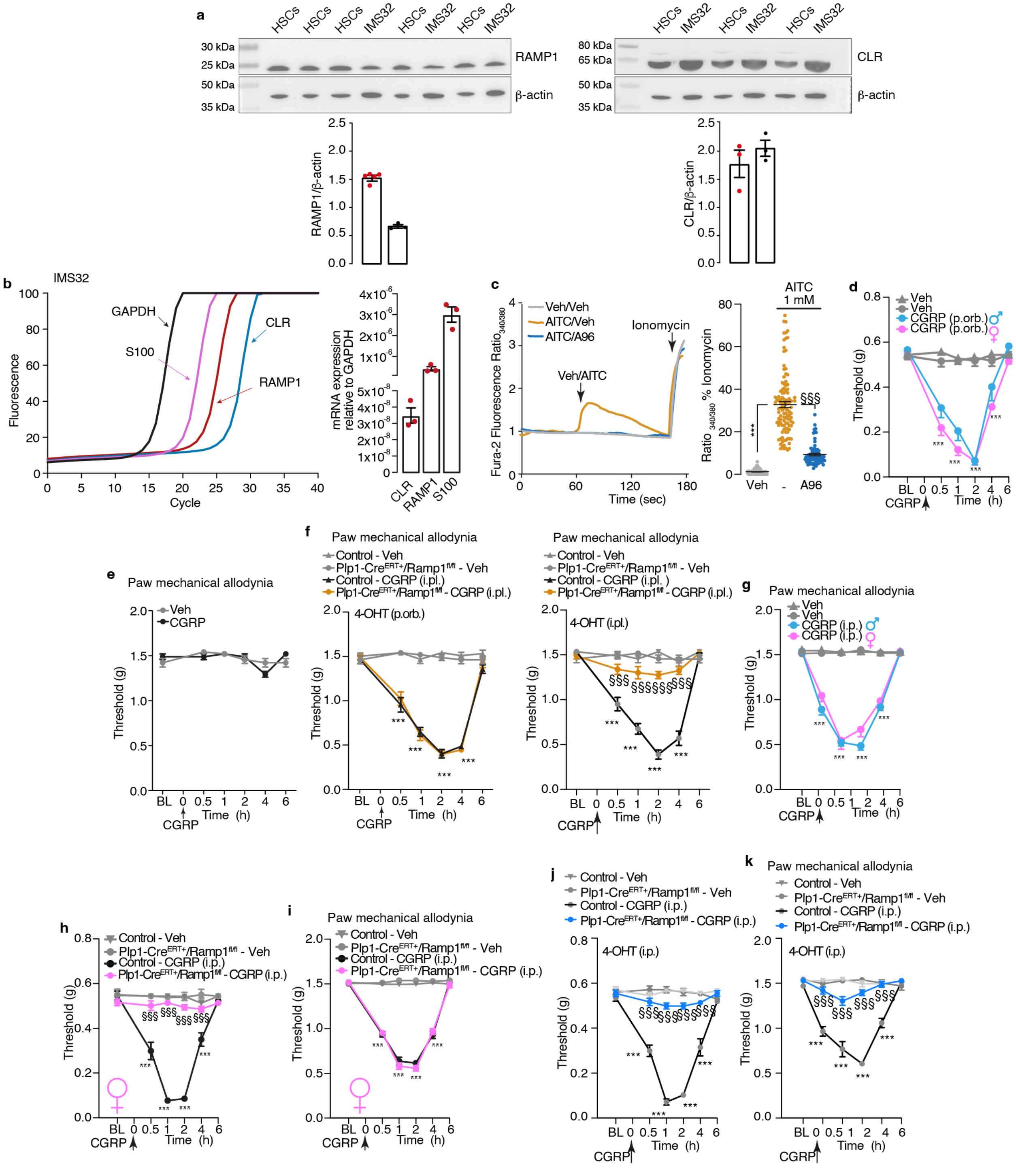

Supplementary Fig. 1. IMS32 characterization and paw allodynia after periorbital treatments. (a) Representative blot and protein content in HSCs and IMS32 cells of RAMP1, CLR and S100. Equal protein loading was verified by expression of β-actin (HSCs RAMP1 n=5, IMS32 RAMP1 n=3, HSCs CLR n=3, IMS32 CLR n=3 independent experiments). (b) Representative real-time PCR plot and cumulative data for GAPDH, S100, CLR and RAMP1 in IMS32 cells (n=3 independent experiments). (c) Representative traces and pooled data of the Ca^{2+} response in IMS32 cells exposed to AITC (30 μM) in the presence of A967079 (A96, 50 μM) or vehicle (Veh n=112, AITC n=112, A96 n=71 cells from n=4 independent experiments). (d) PMA induced by CGRP (1.5 nmol) or vehicle in C57BL/6J male and female mice (n=8 mice per group). (e) Paw mechanical allodynia after periorbital injection of CGRP (1.5 nmol) or vehicle in C57BL/6J mice (n=8 mice per group). (f) Paw mechanical allodynia after intraplantar (i.pl.) injection of CGRP (1.5 nmol) or vehicle in *Plp1-Cre$^{ERT+/}$Ramp1$^{fl/fl}$* or Control mice treated with periorbital or intraplantar 4-OHT (n=8 mice per group). (g) Paw mechanical allodynia induced by intraperitoneal (i.p.) CGRP (0.1 mg/kg) or vehicle in male and female C57BL/6J mice (n=8 mice per group). (h) PMA and (i) paw mechanical allodynia induced by intraperitoneal (i.p.) CGRP (0.1 mg/kg) or vehicle in male and female *Plp1-Cre$^{ERT+/}$Ramp1$^{fl/fl}$* and Control mice treated with periorbital 4-OHT (n=8 mice per group). (j) PMA and (k) paw mechanical allodynia induced by intraperitoneal (i.p.) CGRP (0.1 mg/kg) or vehicle in *Plp1-Cre$^{ERT+/}$Ramp1$^{fl/fl}$* and Control mice treated with intraperitoneal 4-OHT (n=8 mice per group). Mean±SEM. (-) in c represents vehicle of A96. ***P<0.001 vs. Veh/Veh, Veh and Control-Veh, §§§P<0.001 vs. AITC and Control-CGRP. 1-way (c) and 2-way (d-k) ANOVA, Bonferroni correction. Source data are provided as a Source Data file.

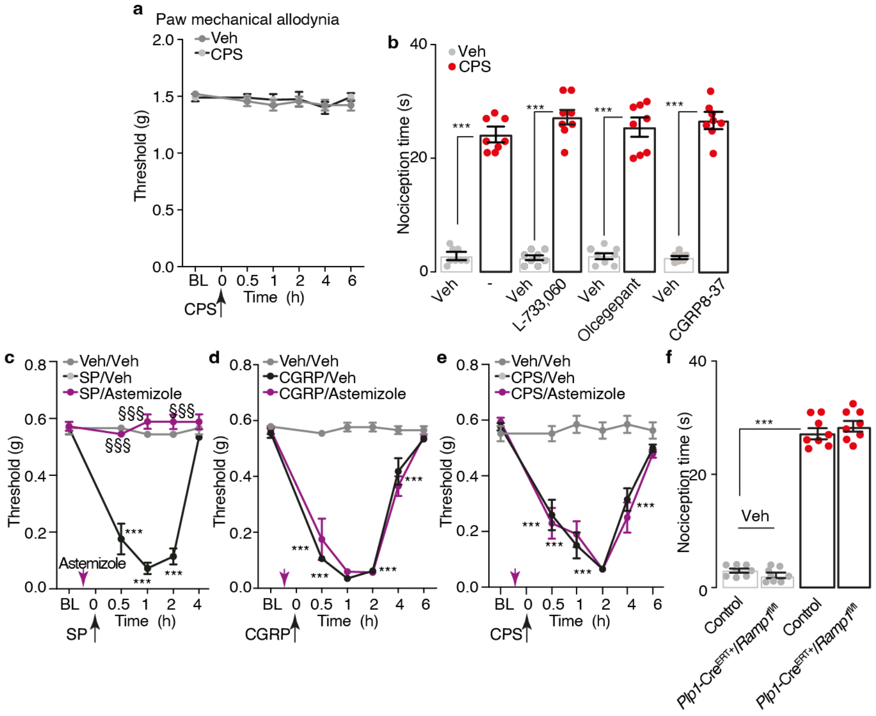

Supplementary Fig. 2. Mechanisms of capsaicin and SP-evoked allodynia. (a) Paw mechanical allodynia after periorbital capsaicin (CPS, 50 pmol) or vehicle in C57BL/6J mice. (b) Acute nociception after periorbital CPS (50 pmol) or vehicle in C57BL/6J mice pretreated with L-733,060 (20 nmol), olcegepant (1 nmol), CGRP8-37 (10 nmol) or vehicles. (c-e) PMA induced by periorbital SP (3.5 nmol), CGRP (1.5 nmol), CPS (50 pmol) or vehicles in C57BL/6J mice pretreated with astemizole (10 nmol). (f) Acute nociceptive response after CPS (50 pmol) or vehicle in *Plp1-Cre*$^{ERT+/}$*Ramp1*$^{fl/fl}$ or Control mice treated with 4-OHT. Mean±SEM. (n=8 mice per group). ***P<0.001 vs. Veh, Veh/Veh and Control-Veh. §§§P<0.001 vs. SP/Veh. Student's t test (b), 1-way (f) or 2-way (c-e) ANOVA, Bonferroni correction. Source data are provided as a Source Data file.

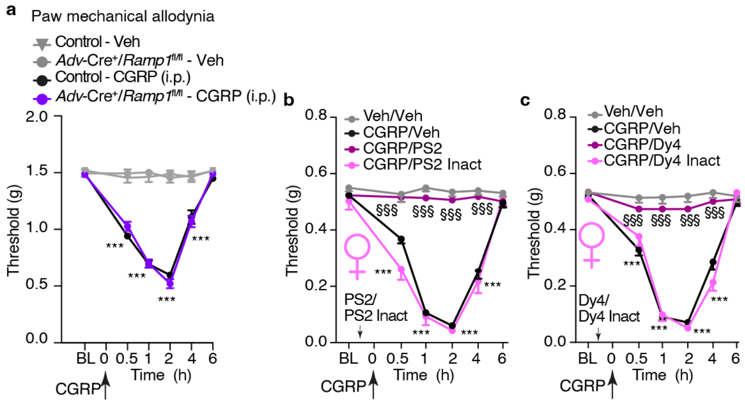

Supplementary Fig. 3. Mechanism of CGRP-evoked allodynia. (a) Paw mechanical allodynia induced by intraperitoneal (i.p.) CGRP (0.1 mg/kg) or vehicle in *Adv-Cre*$^{+/}$*Ramp1*$^{fl/fl}$ or Control mice. (b,c) PMA induced by periorbital CGRP (1.5 nmol) or vehicle in C57BL/6J female mice pretreated (0.5 h) with Dyngo-4a (Dy4), Pitstop 2 (PS2), inactive analogs (PS2 and Dy4 inact) (all 500 pmol) (n=8 mice per group). Mean±SEM. ***P<0.001 vs. Control-Veh and Veh/Veh; §§§P<0.001 vs. CGRP/PS2 inact, CGRP/Dy4 inact. 2-way (a-c) ANOVA, Bonferroni correction. Source data are provided as a Source Data file.

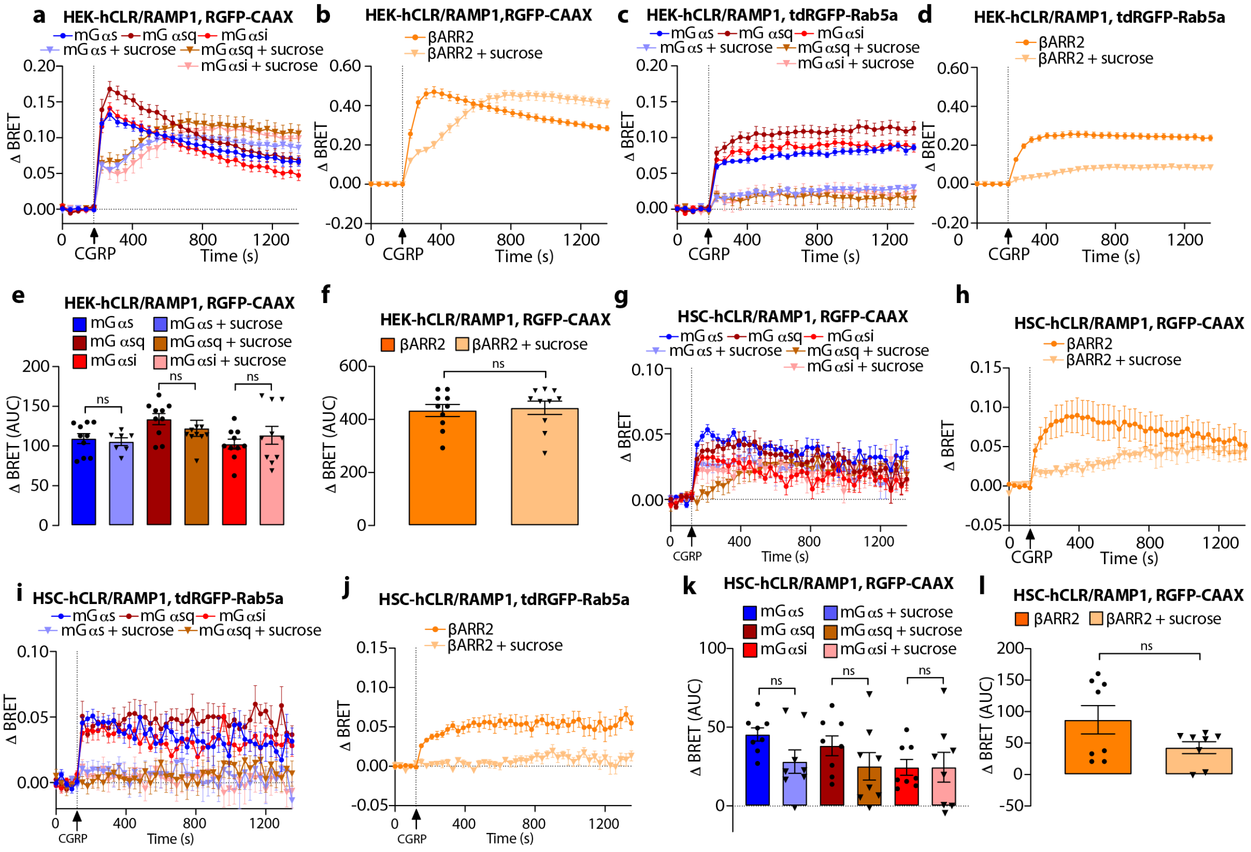

Supplementary Fig. 4. CGRP-induced recruitment of mGα proteins and βARR2 to the plasma membrane and early endosomes in HEK-hCLR/RAMP1 cells and HSC-hCLR/RAMP1 cells. (a-f) Effects of hypertonic (0.45 M) sucrose on CGRP (100 nM)-stimulated EbBRET between Rluc8-mGα_s, Rluc8-mGα_{sq}, Rluc8-mGα_{si}, and Rluc2-βARR2 with RGFP-CAAX (a, b, e, f) and tdRGFP-Rab5a (c, d) in HEK-hCLR/RAMP1 cells. a-d, time course. e, f, area under curve (AUC) (a,e n=10 mGα_s, mGα_{sq}, mGα_{sq} + sucrose, mGα_{si}, mGα_{si} + sucrose, n=7 mGα_s + sucrose independent experiments; b,c,d,f, n=10 independent experiments). (g-l) Effects of hypertonic (0.45 M) sucrose on CGRP (100 nM)-stimulated EbBRET between Rluc8-mGα_s, Rluc8-mGα_{sq}, Rluc8-mGα_{si} and Rluc2-βARR2 with RGFP-CAAX (g, h, k, l) and tdRGFP-Rab5a (i, j) in HSC-hCLR/RAMP1 cells g-j, time course. k, l, AUC (g-l, n=8 independent experiments). Mean±SEM. ns, not significant, parametric unpaired t test. Source data are provided as a Source Data file.

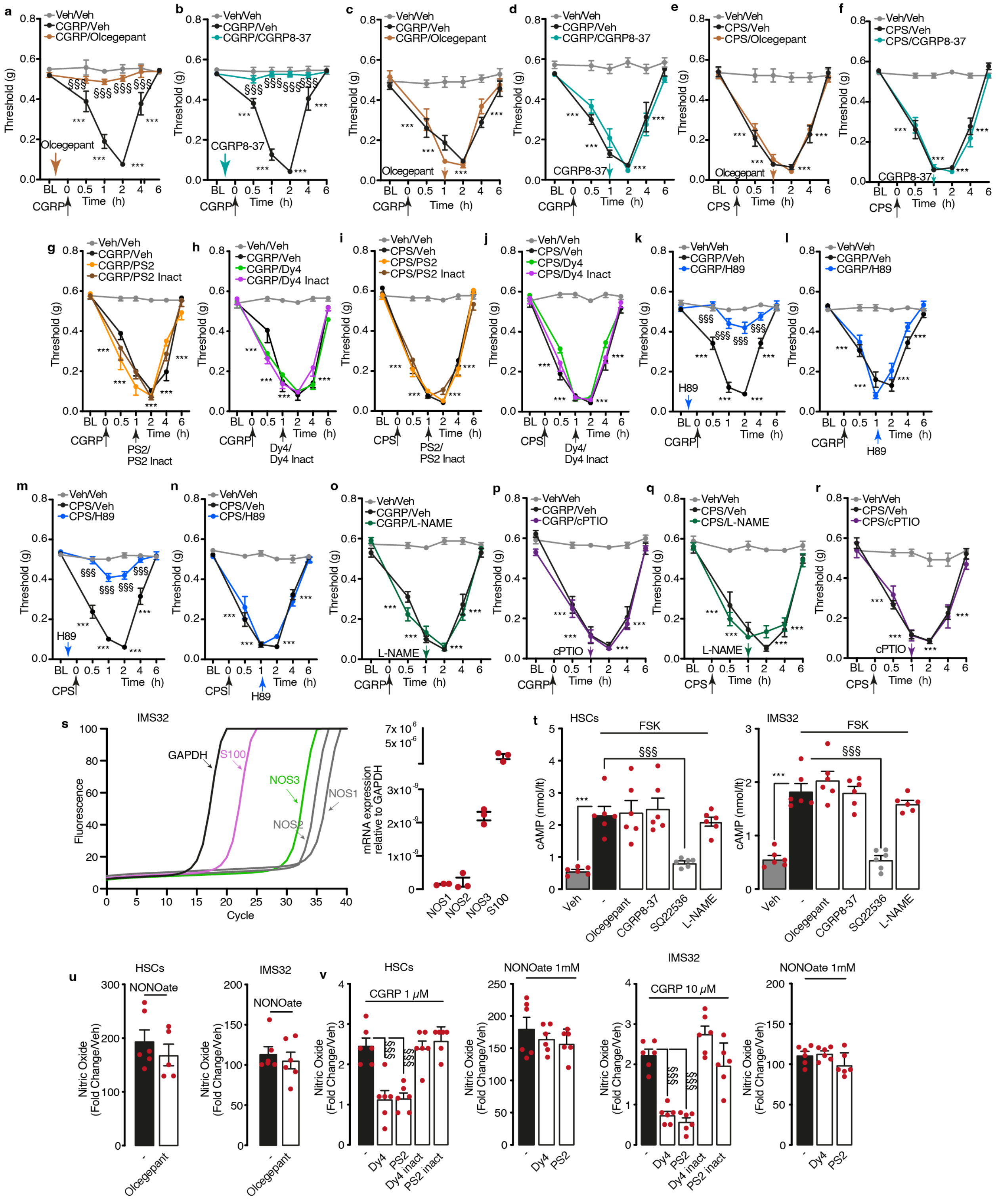

Supplementary Fig. 5. Mechanism of CGRP- and capsaicin-evoked allodynia. (a-n) PMA after CGRP (1.5 nmol), capsaicin (CPS, 50 pmol) or vehicles in C57BL/6J mice pre-treated (30 min) or post-treated (60 min) with olcegepant (1 nmol), CGRP8-37 (10 nmol), Dyngo-4a (Dy4, 500

pmol), Pitstop 2 (PS2, 500 pmol) or inactive analogs (PS2 and Dy4 inact), H89 (45 nmol) or vehicles (n=8 mice per group). (o-r) PMA after CGRP (1.5 nmol), CPS (50 pmol) or vehicles in C57BL/6J mice post-treated (60 min) with L-NAME (1 µmol), cPTIO (200 nmol) or vehicle (n=8 mice per group). (s) Representative real-time PCR plot and cumulative data for GAPDH, S100, NOS1, NOS2 and NOS3 in IMS32 cells (n=3 independent experiments). (t) cAMP ELISA in HSCs and IMS32 cells exposed to forskolin (FSK) or vehicle, and in the presence of olcegepant (100 nM), CGRP8-37 (100 nM), SQ22536 (100 µM), L-NAME (10 µM) or vehicle (n=6 independent experiments). (u) Nitric oxide assay in HSCs and IMS32 cells exposed to NONOate or vehicle, and in the presence of olcegepant (100 nM) or vehicle (n=6 independent experiments). (v) Nitric oxide assay in HSCs and IMS32 cells exposed to CGRP or vehicle, and NONOate or vehicle in presence of Dy4, PS2 or PS2 inact and Dy4 inact (n=6 independent experiments). Mean±SEM. (-) dash represents the combination of different vehicles. ***P<0.001 vs. Veh/Veh and Veh; §§§P<0.001 vs. CGRP/Veh, CPS/Veh and FSK CGRP 1 µM and 10 µM. 2-way (a-r) or 1-way (t-v) ANOVA, Bonferroni correction. Source data are provided as a Source Data file.

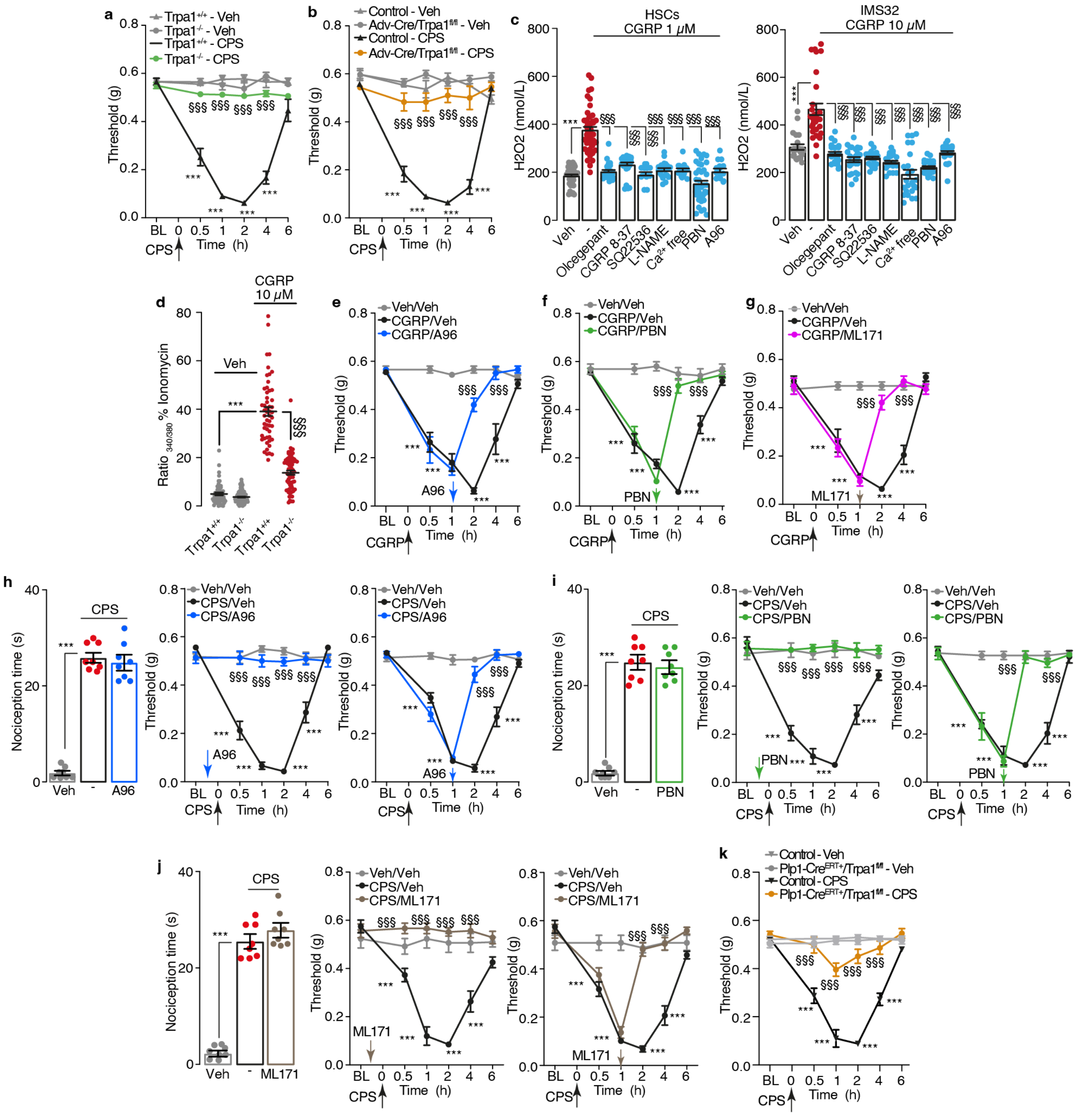

Supplementary Fig. 6. TRPA1 implication in CGRP- and capsaicin-evoked responses. PMA after periorbital injection of capsaicin (CPS, 50 pmol) or vehicle in (a) *Trpa1*$^{+/+}$ and *Trpa1*$^{-/-}$ mice and in (b) *Adv-Cre*$^{+}$/*Trpa1*$^{fl/fl}$ or Control mice (n=8 mice per group). (c) H_2O_2 release in HSCs and IMS32 cells exposed to CGRP (10 µM) in the presence of olcegepant (100 nM), CGRP8-37 (100 nM), SQ22536 (100 µM), L-NAME (10 µM), Ca^{2+}-free medium, PBN (50 µM), A96 (50 µM), or vehicle (0.1 % DMSO) (HSCs and IMS32: veh, n=40 and n=15; CGRP 1 µM, n=47; CGRP 10 µM n=25; olcegepant, n=29 and n=18; CGRP8-37, n=30 and n=22; SQ22536, n=16 and n=18; L-NAME, n=23 and n=16; Ca^{2+}-free medium, n=16 and n=24; PBN, n=16 and n=17; A96, n=36 and n=18 independent experiments, respectively). (d) Ca^{2+} response to CGRP or vehicle in primary

culture of Schwann cells derived from *Trpa1*$^{+/+}$ and *Trpa1*$^{-/-}$ mice (veh/*Trpa1*$^{+/+}$ n=53, veh/*Trpa1*$^{-/-}$ n=141, CGRP/*Trpa1*$^{+/+}$ n=53, CGRP/*Trpa1*$^{-/-}$ n=69 cells). (e-g) PMA after CGRP (1.5 nmol) or vehicle in C57BL/6J male mice post-treated (60 min) with A967079 (A96, 300 nmol), PBN (670 nmol), ML171 (50 nmol) or vehicle (n=8 mice per group). (h-j) Acute nociception and PMA after capsaicin (CPS, 50 pmol) or vehicle in C57BL/6J mice pre-treated (30 min, left) or post-treated (60 min, right) with A96 (300 nmol), PBN (670 nmol), ML171 (50 nmol) or vehicle (n=8 mice per group). (k) PMA after CPS (50 pmol) or vehicles in *Plp1-Cre*$^{ERT+/}$*Trpa1*$^{fl/fl}$ or Control mice treated with 4-OHT (n=8 mice per group). Mean±SEM. (-) dash represents the combination of different veh. ***P<0.001 vs. *Trpa1*$^{+/+}$ Veh, Veh and Veh/Veh, Control/veh; §§§P<0.001 vs. *Trpa1*$^{+/+}$-CPS, Control-CPS, CGRP 1 µM and 10 µM, CGRP/Veh, CPS/Veh. 1-way (c,d, h-j) or 2-way (a,b, e,-k) ANOVA, Bonferroni correction. Source data are provided as a Source Data file.

a

Polymer	Conversion	GPC		^{1}H-NMR				
	(%)	Mn (g/mol)	PDI	Mn (g/mol)	Composition PEGMA	DMAEMA	DIPMA	DEGMA
P(PEGMA -co-DMAEMA)	83	12,500	1.14	17,443	55	6	-	-
P(PEGMA -co-DMAEMA) -b-(DIPMA -co-DEGMA)	92	20,000	1.23	40,319	55	6	94	15
P(PEGMA -co-DMAEMA) -b-(DIPMA -co-DEGMA -co-VDM)	43	19,380	1.22	40,600	56	6	111	12

b

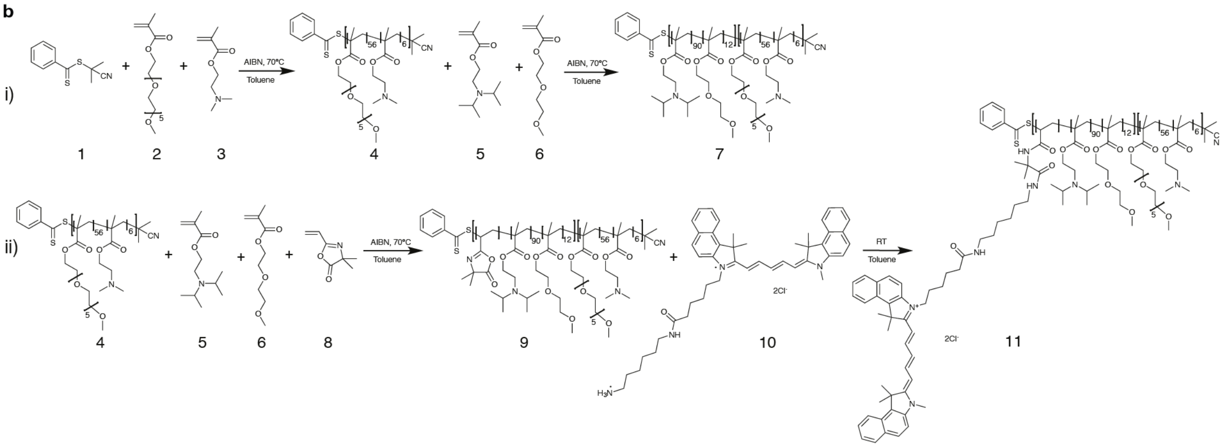

c

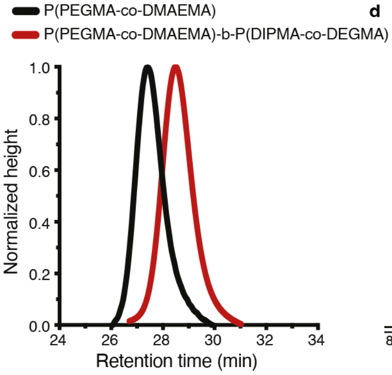

d

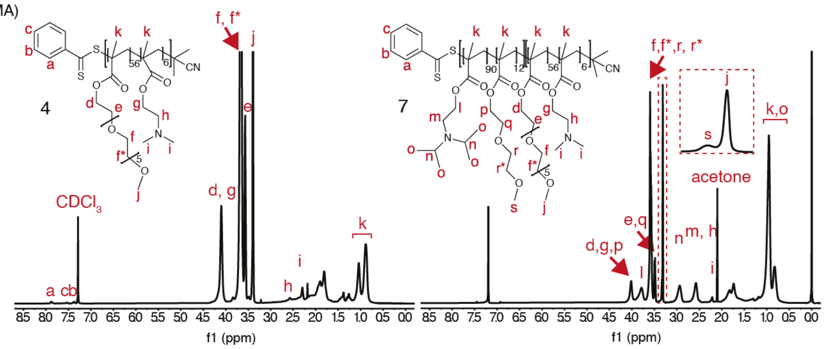

Supplementary Fig. 7. Synthesis and characterization of P(PEGMA-co-DMAEMA)-block-P(DIPMA-co-DEGMA). (a). Characterization of the hydrophilic block copolymers and the diblock copolymers by ^{1}H-NMR and gel permeation chromatography. (b). RAFT polymerization indicating synthesis of the (i) hydrophilic block using (1) CPDB, (2) PEGMA and (3) DMAEMA to form (4) p(PEGMA-co-DMAEMA) and the synthesis of P(PEGMA-co-DMAEMA)-b-P(DIPMA-co-DEGMA) by the subsequent addition of the pH-responsive monomer (5) DIPMA and the charge screening monomer (6) DEGMA to (4) the hydrophilic block to form (7) the diblock P(PEGMA-co-DMAEMA)-b-P(DIPMA-co-DEGMA); ii) Addition of (5) DIPMA, (6) DEGMA and (8) VDM to (4) the hydrophilic block to form the intermediate (9), followed by the addition of (10) Cy5 to form the final Cy5 conjugated polymer (11) P(PEGMA-co-DMAEMA)-b-P(DIPMA-co-DEGMA-co-Cy5). (c) Gel permeation chromatography traces showing a shift from the hydrophilic block P(PEGMA-co-DMAEMA) to higher molecular weight after chain extension to form P(PEGMA-co-DMAEMA)-b-P(DIPMA-co-DEGMA). (d). ^{1}H-NMR spectra of the resulting polymers indicating the successful incorporation of the monomers indicated in b. Source data are provided as a Source Data file

Supplementary Tables

Supplementary Table 1. Pharmacological agents injected subcutaneously in the periorbital area (10 μl/site)

**Agent**	**Target**	**Dose**
CGRP	CLR/RAMP1 Agonist	1.5 nmol
SP	NK1 Agonist	3.5 nmol
Capsaicin	TRPV1 Agonist	10, 50, 100 pmol
Capsazepine	TRPV1 Antagonist	100 pmol
L-733,060	NK1 Antagonist	20 nmol
Astemizole	H_1 Antihistaminic	10 nmol
Olcegepant	CLR/RAMP1 Antagonist	1 nmol
L-NAME	NOS Inhibitor	1 μmol
cPTIO	NO Scavenger	200 nmol
H89	PKA inhibitor	45 nmol
A967079	TRPA1 Antagonist	300 nmol
PBN	Spin trap	670 nmol
ML-171	NOX1 Inhibitor	250 nmol
CGRP8-37	CLR/RAMP1 Antagonist	10 nmol
PitStop2	Clathrin Inhibitor	500 pmol
Dyngo4a	Dynamin Inhibitor	500 pmol
MK-3207	CLR/RAMP1 Antagonist	0.1, 0.3, 1 pmol
DIPMA-MK-3207	Nanoparticles loaded with MK-3207	0.1, 0.3, 1 pmol

*In some experiments CGRP (1.5 nmol) was injected in the mouse hind paw (20 μl/site, intraplantar)

Supplementary Table 2. Pharmacological agents used in cultured cells.

Agent	Target	Concentration
CGRP	CLR/RAMP1 Agonist	1^{-10}-10^{-5} M
TAMRA-CGRP	CLR/RAMP1 Agonist	3.5 nmol
Olcegepant	CLR/RAMP1 Antagonist	10^{-12}-10^{-4} M
MK-3207	CLR/RAMP1 Antagonist	10^{-8}-10^{-3} M
DIPMA-MK-3207	Nanoparticles loaded with MK-3207	10^{-9}-10^{-7} M
PitStop2	Clathrin Inhibitor	30 μM
Dyngo4a	Dynamin Inhibitor	30 μM
Sucrose	Clathrin-mediated endocytosis inhibitor	0.45 M
A967079	TRPA1 Antagonist	50 μM
L-NAME	NOS Inhibitor	10 μM
PBN	Spin trap	50 μM
H89	PKA inhibitor	1 μM
ML-171	NOX1 Inhibitor	1 μM
CGRP8-37	CLR/RAMP1 Antagonist	100 nM
SQ22536	Adenylyl cyclase inhibitor	100 μM
Forskolin	Adenylyl cyclase activator	1-10 μM
NONOate	NO donor	1 mM

Supplementary Table 3. Human and mouse primers used in this study, related to qRT-PCR.

	Sequence (5' to 3')
Human Gapdh (NM_002046)	F: ACATCGCTCAGACACCATG<br>R: TGTAGTTGAGGTCAATGAAGGG
Human S100 (NM_006272)	F: CACAAGCTGAAGAAATCCGAAC<br>R: CACATTCGCCGTCTCCATC
Human Ramp1 (NM_005855)	F: ACCCAGTTCCAGGTAGACAT<br>R: CAGCTTCTCCGCCATGTG
Human Clr (NM_005795)	F: GTAACAATCATTCACCTCA CTGC<br>R: GCCTTCACAGAGCATCCAA
Human Nos1 (NM_001204218)	F: TCAACTACATCTGTAACCACGTC<br>R: AGTCATGCTTGCCATCAGTC
Human Nos2 (NM_000625)	F: GACTGAGCTGTTAGAGACACTT<br>R: CACTTCTGCTCCAAATCCAAC
Human Nos3 (NM_000603)	F: CTTGAGGATGTGGCTGTGT<br>R: TGGTCCACTATGGTCACTTTG
Mouse Gapdh (NM_008084)	F: AATGGTGAAGGTCGGTGTG<br>R:GTGGAGTCATACTGGAACATGTAG
Mouse S100 (NM_011309)	F: TGGATGAAAACGGAGATGGGG<br>R: ACAGACTGTGCTCAACTGGT
Mouse Ramp1 (NM_178401)	F: GGGGCTCTGCTTGCCAT<br>R: GGATGAGAGTCCCATAGTCAGG
Mouse Clr (NM_018782)	F: TTTCTGGTTCTCTTGCCTCTT<br>R: ATTGAGCCGTCATGATCTTGT
Mouse Nos1 (NM_008712)	F: TCAACTACATCTGTAACCACGTC<br>R: AGTCATGCTTGCCATCAGTC
Mouse Nos2 (NM_010927)	F: GACTGAGCTGTTAGAGACACTT<br>R: CACTTCTGCTCCAAATCCAAC
Mouse Nos3 (NM_008713)	F: CTTGAGGATGTGGCTGTGT<br>R: TGGTCCACTATGGTCACTTTG
