# Supplementary material for: Schwann cell endosome CGRP signals elicit periorbital mechanical allodynia in mice
Source: Nat Commun. 2022 Feb 3;13:646. doi: 10.1038/s41467-022-28204-z (PMC8813987; doi:10.1038/s41467-022-28204-z)
Supplement: Supplementary file 2 — Description of Additional Supplementary Files [file 41467_2022_28204_MOESM2_ESM.docx]

Description of Additional Supplementary Files

**Title: Supplementary Video 1. CGRP-evoked cAMP responses in HSCs.**

**Description:** cAMP was measured using the CADIS assay. Inset shows cAMP signals in individual cells denoted by regions of interest (ROI) 1-6. Cells were challenged with 100 nM CGRP followed by 1 µM isoproterenol.

**Title: Supplementary Video 2. Uptake of TAMRA-CGRP in HSCs expressing Rab5a-GFP.**

**Description:** Left panel, TAMRA-CGRP. Right panel, TAMRA-CGRP merged with Rab5a-GFP (endosomes).

**Title: Supplementary Video 3. Inhibition of uptake of TAMRA-CGRP in PS2-treated HSCs expressing Rab5a-GFP.**

**Description:** Left panel, TAMRA-CGRP. Right panel, TAMRA-CGRP merged with Rab5a-GFP (endosomes).

**Title: Supplementary Video 4. Inhibition of uptake of TAMRA-CGRP in Dy4-treated HSCs expressing Rab5a-GFP.**

**Description:** Left panel, TAMRA-CGRP. Right panel, TAMRA-CGRP merged with Rab5a-GFP (endosomes).

**Title: Supplementary Video 5. Inhibition of uptake of TAMRA-CGRP in sucrose-treated HSCs expressing Rab5a-GFP.**

**Description:** Left panel, TAMRA-CGRP. Right panel, TAMRA-CGRP merged with Rab5a-GFP (endosomes).

**Title: Supplementary Video 6. Uptake of TAMRA-CGRP in HSCs expressing EEA1-GFP pretreated with DIPMA-Cy5.**

**Description:** Left panel, TAMRA-CGRP. Right panel, TAMRA-CGRP merged with DIPMA-Cy5 and EEA1-GFP (endosomes).

Title: Supplementary data 1

Description: Supplementary Data file reports detailed statistics from each figure.
