# Supplementary material for: Schwann cell endosome CGRP signals elicit periorbital mechanical allodynia in mice
Source: Nat Commun. 2022 Feb 3;13:646. doi: 10.1038/s41467-022-28204-z (PMC8813987; doi:10.1038/s41467-022-28204-z)
Supplement: Supplementary file 10 — Reporting Summary [file 41467_2022_28204_MOESM10_ESM.pdf]

nature portfolio

Corresponding author(s): Pierangelo Geppetti, Nigel W Bunnett

Last updated by author(s): Dec 22, 2021

Reporting Summary

Nature Portfolio wishes to improve the reproducibility of the work that we publish. This form provides structure for consistency and transparency in reporting. For further information on Nature Portfolio policies, see our Editorial Policies and the Editorial Policy Checklist.

Statistics

For all statistical analyses, confirm that the following items are present in the figure legend, table legend, main text, or Methods section.

n/a	Confirmed	
☐	☒	The exact sample size (*n*) for each experimental group/condition, given as a discrete number and unit of measurement
☐	☒	A statement on whether measurements were taken from distinct samples or whether the same sample was measured repeatedly
☐	☒	The statistical test(s) used AND whether they are one- or two-sided<br>*Only common tests should be described solely by name; describe more complex techniques in the Methods section.*
☐	☒	A description of all covariates tested
☐	☒	A description of any assumptions or corrections, such as tests of normality and adjustment for multiple comparisons
☐	☒	A full description of the statistical parameters including central tendency (e.g. means) or other basic estimates (e.g. regression coefficient) AND variation (e.g. standard deviation) or associated estimates of uncertainty (e.g. confidence intervals)
☐	☒	For null hypothesis testing, the test statistic (e.g. *F*, *t*, *r*) with confidence intervals, effect sizes, degrees of freedom and *P* value noted<br>*Give P values as exact values whenever suitable.*
☒	☐	For Bayesian analysis, information on the choice of priors and Markov chain Monte Carlo settings
☒	☐	For hierarchical and complex designs, identification of the appropriate level for tests and full reporting of outcomes
☐	☒	Estimates of effect sizes (e.g. Cohen's *d*, Pearson's *r*), indicating how they were calculated

Our web collection on statistics for biologists contains articles on many of the points above.

Software and code

Policy information about availability of computer code

Data collection	Plate reader Flex station III, SoftMax Pro 7 Data Acquisition and Analysis Software<br>Zeiss Axio Imager 2, Zeiss ZEN imaging (2020)<br>Leica SP8 confocal microscope, imaging software LAS Version X<br>ChemiDoc Imaging System, Bio-Rad Version 2.3.0.07<br>Dynamic light scattering Zetasizer Nano ZS ZEN3600 particle size analyser<br>TEM imaging Tecnai F20 transmission electron microscope<br>Olympous microscope IX 81
Data analysis	ImageJ 1.32J software NIH https://imagej.nih.gov/<br>G*Power (v3.1) https://www.psychologie.hhu.de/arbeitsgruppen/allgemeine-psychologie-und-arbeitspsychologie/gpower.html<br>Research Randomizer https://www.randomizer.org/<br>GraphPad Prism 8, Software Inc. https://www.graphpad.com/scientific-software/prism/<br>Plate reader Flex station III, SoftMax Pro 7 Data Acquisition and Analysis Software<br>Rotor-Gene ® Q - Software Version 2.3.1.49<br>ChemiDoc Imaging System, Bio-Rad Version 2.3.0.07

For manuscripts utilizing custom algorithms or software that are central to the research but not yet described in published literature, software must be made available to editors and reviewers. We strongly encourage code deposition in a community repository (e.g. GitHub). See the Nature Portfolio guidelines for submitting code & software for further information.

Data

Policy information about availability of data

All manuscripts must include a data availability statement. This statement should provide the following information, where applicable:

- Accession codes, unique identifiers, or web links for publicly available datasets
- A description of any restrictions on data availability
- For clinical datasets or third party data, please ensure that the statement adheres to our policy

Source data file has been provided

Field-specific reporting

Please select the one below that is the best fit for your research. If you are not sure, read the appropriate sections before making your selection.

- [x] Life sciences
- [ ] Behavioural & social sciences
- [ ] Ecological, evolutionary & environmental sciences

For a reference copy of the document with all sections, see nature.com/documents/nr-reporting-summary-flat.pdf

Life sciences study design

All studies must disclose on these points even when the disclosure is negative.

Sample size	The group size of n = 8 animals for behavioral experiments was determined by sample size estimation using G*Power (v3.1) to detect size effect in a post-hoc test with type 1 and 2 error rates of 5 and 20%, respectively.<br>No sample size calculations were performed for in vitro experiments. The sample size (n) of each experiment is provided in the corresponding figure captions in the main manuscript and supplementary information files. Sample sizes were chosen to support meaningful conclusions.
Data exclusions	No animals were excluded from experiments
Replication	Experimental findings were reliably reproduced among all experiments. The detailed information is reported throughout the figure legends.
Randomization	Mice were allocated to vehicle or treatment groups using a randomization procedure (http://www.randomizer.org/). Random allocation was maintained throughout the study.
Blinding	4 independent and blinded investigators performed the treatments, behavioral experiments, genotyping and data analysis respectively.

Reporting for specific materials, systems and methods

We require information from authors about some types of materials, experimental systems and methods used in many studies. Here, indicate whether each material, system or method listed is relevant to your study. If you are not sure if a list item applies to your research, read the appropriate section before selecting a response.

Materials & experimental systems

n/a	Involved in the study	
[ ]	[x]	Antibodies
[ ]	[x]	Eukaryotic cell lines
[x]	[ ]	Palaeontology and archaeology
[ ]	[x]	Animals and other organisms
[ ]	[x]	Human research participants
[x]	[ ]	Clinical data
[x]	[ ]	Dual use research of concern

Methods

n/a	Involved in the study	
[x]	[ ]	ChIP-seq
[x]	[ ]	Flow cytometry
[x]	[ ]	MRI-based neuroimaging

Antibodies

Antibodies used	1.Rabbit monoclonal anti-RAMP1 [ERP10867] Abcam Cat#ab156575 Lot: GR3196403-5<br>2.Rabbit polyclonal anti-S100 Abcam Cat#ab34686 Lot: GR64881-17<br>3.Rabbit polyclonal anti-CRLR Novus Biologicals Cat#NBP1-59073 Lot: QC58878-190422<br>4.Mouse monoclonal anti-b-actin Abcam [AC-15] Cat#ab6276 Lot: GR 141-7<br>5.Goat anti-mouse IgG conjugated with horseradish peroxidase (HRP) Bethyl Laboratories Inc Cat#A90-516P<br>6.Donkey anti-rabbit IgG conjugated with horseradish peroxidase (HRP) Bethyl Laboratories Inc Cat#A120-208P<br>7.Rabbit polyclonal anti-eNOSpS1177 Abcam Cat#ab184154 Lot: GR3257047-9<br>8.Rabbit polyclonal anti-TRPA1 Abcam Cat#ab58844 Lot: GR165165-21

	9.Mouse monoclonal anti-S100 [4B3] Abcam Cat#ab14849 Lot: GR3233892-2<br>10.Rabbit polyclonal anti-CRLR Novus Biologicals Cat#NLS6731 Lot: 8312<br>11.Rabbit polyclonal anti-RAMP1 Alexa Fluor 594 Abcam Cat#ab241335 Lot: GR3247267-4<br>12.Goat anti-rabbit IgG (H+L) Highly Cross-Adsorbed Secondary Antibody Alexa Fluor Plus 488 Thermo Fischer Scientific Cat#A32731 Lot: VA295501<br>13.Goat anti-mouse IgG (H+L) Highly Cross-Adsorbed Secondary Antibody Alexa Fluor Plus 555 Thermo Fischer Scientific Cat#A32727 Lot: UL287768
Validation	1. Rabbit monoclonal anti-RAMP1 Abcam Cat#ab156575 (doi: 10.1210/jendso/bvaa199)<br>2. Rabbit polyclonal anti-S100 Abcam Cat#ab34686 (doi: 10.18632/oncotarget.9618)<br>3. Rabbit polyclonal anti-CRLR Novus Biological Cat#NBP1-59073 (https://www.novusbio.com/products/crlr-antibody_nbp1-59073)<br>4. Mouse monoclonal anti-β-actin Abcam Cat#ab6276 (doi: 10.1186/s13578-020-00422-2)<br>5. Goat anti-mouse IgG conjugated with horseradish peroxidase (HRP) Bethyl Laboratories Inc Cat#A90-516P (doi: 10.1038/s41467-020-20826-5)<br>6. Donkey anti-rabbit IgG conjugated with horseradish peroxidase (HRP) Bethyl Laboratories Inc Cat#A120-208P (doi:10.1007/s00280-014-2386-z)<br>7. Rabbit polyclonal anti-eNOSpS1177 Abcam Cat#ab184154 (doi: 10.1002/jcp.28969)<br>8. Rabbit polyclonal anti-TRPA1 Abcam Cat#ab58844 (doi: 10.1172/JCI128022; doi: 10.1113/jphysiol.2011.206789; doi: 10.1038/s41598-019-55133-7)<br>9. Mouse monoclonal anti-S100 Abcam Cat#ab14849 (doi:10.1111/cpr.12756)<br>10. Rabbit polyclonal anti-CRLR Novus Biologicals Cat#NLS6731 (https://www.novusbio.com/products/crlr-antibody_nls6731)<br>11. Rabbit polyclonal anti-RAMP1 Abcam Cat#ab241335 (https://www.abcam.com/alexa-fluor-594-ramp1-antibody-ab241335.html)<br>12. Polyclonal secondary antibodies Alexa Fluor 488 Thermo Fisher Scientific Cat#A32731 (doi: 10.1016/j.celrep.2018.11.090)<br>13.Polyclonal secondary antibodies Alexa Fluor 555 Thermo Fisher Scientific Cat#A32727 (doi: 10.1186/s40478-018-0528-y)

Eukaryotic cell lines

Policy information about cell lines

Cell line source(s)	Human Schwann cells ScienCell Research Laboratories Cat#1700<br>Mouse Schwann cell line (IMS32) Cosmo Bio Cat#PMC-SWN-IMS32-COS<br>HEK293T American Type Culture Collection (ATCC) Cat#ATCC™-CRL-3216™
Authentication	All cells were used when received without further authentication.
Mycoplasma contamination	Cell Lines were negative to mycoplasm contamination.
Commonly misidentified lines<br>(See ICLAC register)	No commonly misidentified cell lines were used.

Animals and other organisms

Policy information about studies involving animals; ARRIVE guidelines recommended for reporting animal research

Laboratory animals	C57BL/6J (Charles River, RRID:IMSR_JAX:000664) mice (male, female 25-30 g, 5-8 weeks)<br>TRPA1-deficient (Trpa1-/-; B6129P-Trpa1tm1Kykw/J; RRID:IMSR_JAX:006401, Jackson Laboratory) mice (male, 25-30 g, 5-8 weeks)<br>TRPV1-deficient (Trpv1-/-; B6129X1-Trpv1tm1Jul/J, RRID:IMSR_JAX:003770, Jackson Laboratory) mice (male 25-30 g, 5-8 weeks)<br>129S-Trpa1tm2Kykw/J (floxed TRPA1, Trpa1fl/fl, RRID:IMSR_JAX:008649 Jackson Laboratory) mice (male, female 25-30 g, 5-8 weeks)<br>C57BL/6N-Ramp1<tm1c(EUCOMM)Wtsi>/H (floxed Ramp1, Ramp1fl/fl Stock No: EM:07401, MRC HARWELL Mary Lion Center) mice (male, female 25-30 g, 5-8 weeks)<br>B6.Cg-Tg(Plp1-CreERT)3Pop/J mice (Plp1-CreERT, RRID:IMSR_JAX:005975 Jackson Laboratory) mice (male, female 25-30 g, 5-8 weeks)<br>Advillin-Cre mice (Adv-Cre) mice (male, female 25-30 g, 5-8 weeks) donate by Prof AI Basbaum University of California, US<br>Plp-CreERT+;Ramp1fl/fl and Plp-CreERT-;Ramp1fl/fl (male, female 25-30 g, 5-8 weeks)<br>Adv-Cre+;Ramp1fl/fl and Adv-Cre-;Ramp1fl/fl (male, 25-30 g, 5-8 weeks)<br>Plp1-CreERT;Trpa1fl/fl and Plp1-CreERT-;Trpa1fl/fl (male, 25-30 g, 5-8 weeks)<br>Adv-Cre+;Trpa1fl/fl and Adv-Cre-;Trpa1fl/fl (male, 25-30 g, 5-8 weeks)
Wild animals	No wild animals were used in the study
Field-collected samples	No field collected samples were used in the study
Ethics oversight	All behavioral experiments were in accordance with European Union (EU) guidelines for animal care procedures and the Italian legislation (DLgs 26/2014) application of the EU Directive 2010/63/EU.<br>Study was approved by the National Committee for the Protection of Animals used for Scientific Purposes of the Italian Ministry of Health (research permits #383/2019-PR and #765/2019-PR).

Note that full information on the approval of the study protocol must also be provided in the manuscript.

Human research participants

Policy information about studies involving human research participants

Population characteristics	Biological samples used for the study consisted of fragments of healthy skin tissue (waste tissue) and soft tissue obtained following resection of the skin and soft tissues that will not be usable for histopathological diagnosis (and which would therefore be destroyed after surgery).<br>Patients gave their consent for histological samples to be used for scientific studies prior to surgery. Biopsies of human abdominal skin analyzed derived from 3 different patients [female, median age 58 years (range 56-61 years)].
Recruitment	Inclusion criteria:<br>1. Subjects (men and women) aged > 18 years<br>2. Patients who have undergone skin biopsy/resection following precancerous or cancerous lesions<br>3. Patients who have provided their signed informed consent.
Ethics oversight	The use of formalin fixed paraffin embedded (FFPE) sections of human abdominal cutaneous tissues was approved by the Local Ethics Committee of the Florence University Hospital (Area Vasta Toscana Centro) (18271_bio/2020)

Note that full information on the approval of the study protocol must also be provided in the manuscript.
